# MXene-based materials for oil–water separation: mechanisms, performance, environmental implications, and future directions

**DOI:** 10.1039/d6ra01133k

**Published:** 2026-07-06

**Authors:** Shohreh Azizi

**Affiliations:** a UNESCO-UNISA Africa Chair in Nanosciences and Nanotechnology, College of Graduate Studies, University of South Africa Muckleneuk Ridge, PO Box 392 Pretoria 0002 South Africa azizis@unisa.ac.za

## Abstract

Oil–water contamination from industrial effluents and accidental spills presents a persistent environmental challenge, necessitating efficient and sustainable separation technologies. Two-dimensional MXenes, a class of transition-metal carbides and nitrides, have emerged as highly promising materials for oil–water separation due to their atomically thin layered structures, high electrical conductivity, and abundant surface terminations (–O, –OH, and –F). These intrinsic characteristics impart superhydrophilicity and underwater superoleophobicity, enabling selective water permeation while effectively repelling oil phases. To further enhance separation performance, a wide range of MXene-based architectures have been developed, including polymer–MXene composite membranes, three-dimensional porous hybrids with carbon nanomaterials, and multifunctional composites incorporating metal oxides, biopolymers, or clays to improve mechanical robustness, fouling resistance, and chemical stability. Oil–water separation using MXenes proceeds through synergistic mechanisms such as interlayer nanochannel size-sieving, wettability-controlled selective transport, capillary-driven sorption in aerogels and sponges, photothermal-assisted demulsification of viscous oils, and externally stimulated (magnetic or electro-assisted) oil recovery. This review emphasizes recent advances in MXene-based material design, separation mechanisms, and composite engineering for oil–water remediation, with particular focus on establishing a mechanistic framework that links material properties to separation behavior across different system configurations. By identifying current limitations and future research directions, this review aims to provide a comprehensive framework for the rational development and practical deployment of MXene-enabled oil–water separation technologies.

## Abstract 2

Oil–water contamination from industrial effluents and accidental spills presents a persistent environmental challenge, necessitating efficient and sustainable separation technologies.

## Graphical abstract

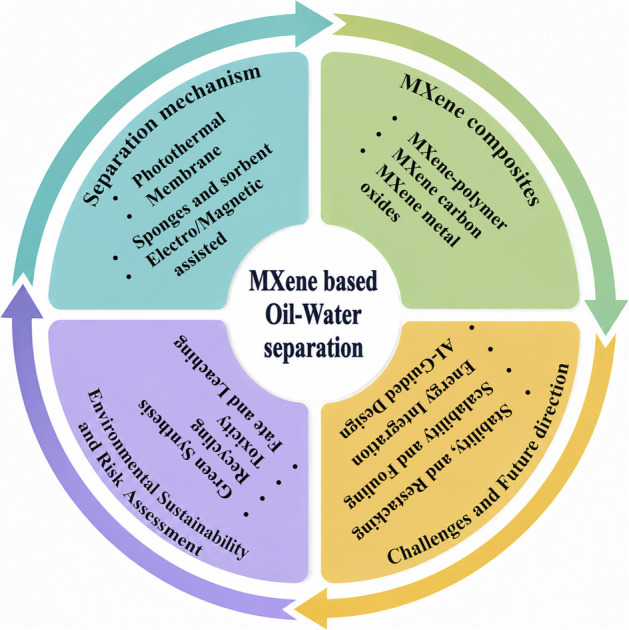

## 1. Introduction

Rapid industrial and social development has increased oil-containing discharge from petrochemical, textile, metallurgical, food and marine industries, leading to severe ecological and health issues.^1–3^ Estimates show that 10–15 billion m^3^ of oily wastewater is produced annually, resulting in more than one million tons of petroleum being discharged into water bodies.^1^ These contaminations worsen water quality, damage aquatic life, and introduce long-lasting hydrocarbons (VOCs, PAHs, *etc.*) into the environment, which cause long-term damage to ecosystems and pose health hazards to the population.^4–6^ The tragic effects of uncontrolled oil discharge were highlighted by high-profile oil spills (*e.g.*, Exxon Valdez and Deepwater Horizon), inspiring the creation of efficient and scalable remediation methods.^7^ Traditional oil–water separation approaches, such as gravity settling, skimming, hydrocyclones, flotation, and chemical coagulation/flocculation, are still commonly used but exhibit inherent limitations.^8^ These methods cannot effectively handle fine (sub-micron) oil droplets or stable emulsions, typically require the use of chemical additives (coagulants and flocculants), consume high amounts of energy, and generate secondary waste streams (*e.g.*, oily sludge).^9,10^ For instance, dissolved-air flotation can only separate oil droplets larger than tens of microns, meaning that smaller emulsions require pretreatment stages.^11^ In addition, flocculation and demulsification mechanisms generate high amounts of polluted biomass or sludge that require different management.^12,13^ Conventional methods are characterized by poor ability to remove emulsified oil, high operational costs, and environmental tradeoffs that limit their sustainability. Membrane filtration has become a new option with significant potential owing to its small modularity and selectivity.^14^ Specially engineered membranes relying on size sieving and wettability effects can in principle retain oil droplets while allowing water to permeate.^15^ Polymeric membranes (PVDF, PS, PP, PTFE, *etc.*) have already been applied for oily wastewater, but their inherently hydrophobic surfaces rapidly foul when exposed to oil, causing dramatic flux decline.^16^ Indeed, the trade-off between permeate flux and oil rejection in these membranes is severe: fouling by oil adsorption or pore clogging typically reduces the recovery flux to only a few percent of the original value.^17,18^ In practice, even ultrafiltration membranes fail to completely remove sub-20 µm droplets without additional pretreatment.^18,19^ Although membrane-based separation offers theoretical advantages (high selectivity, low energy, and no chemicals), in practice it demands new materials with tailored surface chemistry and structure to resist fouling and maintain high throughput. Two-dimensional (2D) nanomaterials have recently gained prominence for oil–water separation because their atomic-scale thickness and stacked layers can form ultrathin sieving channels. MXenes, a family of 2D early transition-metal carbides, nitrides and carbonitrides (M_*n*+1_X_*n*_T_*x*_) have emerged as outstanding candidates for separation membranes and sorbents.^20^ Synthesized by etching the “A” layers from layered MAX phases,^21^ MXenes inherit a high aspect-ratio, metallic conductivity and abundant surface terminations (–OH, –O, and –F).^22,23^ These surface groups render MXenes intrinsically hydrophilic and negatively charged in water, favoring rapid water transport while repelling nonpolar oil species. As a result, MXene films and composites can achieve superhydrophilicity/underwater superoleophobicity, allowing them to wick water through nanoporous channels while rejecting oil droplets.^24^ For example, a Ti_3_C_2_T_*x*_ MXene–paper composite membrane was shown to attain 99% oil-removal efficiency for emulsions, with a water flux exceeding 450 L m^−2^ h^−1^ bar^−1^, due to its highly hydrophilic, tortuous nanochannels.^25^ Besides wettability, the laminated structure of MXenes offers size-selective separation with tunable interlayer spacing (1–2 nm), while their high intrinsic conductivity enables high broadband light absorption.^26^ These features form the basis of photothermal effects: MXene-based materials can rapidly heated under sunlight or NIR irradiation, aiding demulsification and viscous-oil evaporation and enhancing absorption and clean-up performance. Such combined properties provide MXenes with distinct prospects of oil-water separation that are superior to traditional polymers or other 2D materials.^27^ To utilize these opportunities, scientists have synthesized MXenes into various structures suitable in oil remediation. Laminar MXene membranes (freestanding or supported on substrates) take advantage of their natural hydrophilicity to achieve underwater-oil-repellent filtration. Meanwhile, three-dimensional MXene aerogels and sponges are often reinforced with polymers, carbon nanotubes/graphene, or cellulose fibers to provide vast porosity and oil-sorbing capacity. For instance, a recent work developed a cellulose nanofiber–MXene aerogel with hydrophobic coating and embedded gold nanoparticles; this material exhibited high compressive strength, a water contact angle of 145°, and strong photothermal heating (to 76 °C under NIR light), enabling rapid uptake of highly viscous crude oil at 45–85 g oil/g aerogel.^28^ These 3D MXene composites can absorb oil from water surfaces or emulsions by capillary action and can then be readily cleaned by heating or squeezing, offering a reusable and energy-efficient separation approach. In combination, these MXene-based membranes, sorbents and hybrids leverage multi-scale porosity and wettability programming to tackle emulsified, free-oil and surfactant-stabilized mixtures under varying conditions.^29,30^ The escalating problem of oily wastewater discharge and the shortcomings of traditional methods have motivated the exploration of MXene-based materials as next-generation separation media. The intrinsic hydrophilicity, surface charge and photothermal response of MXenes, together with strategies to prevent restacking (*via* intercalation or composite formation), enable high-flux, high-efficiency oil rejection with robust anti-fouling performance.

Despite the growing number of review articles on MXene-based membranes and 2D materials for water treatment, a clear mechanistic and integrative framework for oil–water separation remains lacking. Previous reviews have primarily focused on material synthesis and membrane modification strategies or have discussed MXenes within the broader context of two-dimensional materials. In contrast, the present review aims to establish a mechanism-centered perspective by systematically distinguishing between key separation pathways, including wettability-controlled interfacial rejection, nanochannel-mediated transport, capillary-driven absorption, and externally stimulated processes such as photothermal, magnetic, and electro-assisted separation. Furthermore, this work integrates multiple device configurations (membranes, sorbents, and hybrid systems) within a unified framework, enabling direct comparison of performance-governing mechanisms across different platforms. Importantly, a dedicated section on sustainability, life-cycle impact, and environmental risk is included to address the practical implications of MXene deployment, which remains underexplored in the existing literature. In addition, emerging MXene chemistries beyond Ti_3_C_2_T_*x*_ and the role of surface termination engineering are discussed to provide forward-looking insights. Collectively, these aspects distinguish this review from prior studies and provide a more comprehensive and mechanistically grounded understanding of MXene-enabled oil–water separation.

### 1.1 Properties of MXenes relevant to oil–water separation

MXenes, a fast-growing class of 2D transition metal carbides, nitrides, and carbonitrides, possess various physicochemical characteristics, that make them incredibly suitable for oil-water separation processes.^31,32^ The inherent hydrophilicity of MXenes is one of their most distinct properties, which is primarily determined by the large number of surface hydroxyl and oxygen terminations.^33,34^ This hydrophilicity facilitates water permeation through MXene-based membranes and promotes the repulsion of non-polar organic solvents and oils. Furthermore, the tunable surface chemistry of MXenes enables the selective separation of oil-in-water (O/W) and water-in-oil (W/O) emulsions. This can be achieved through post-synthetic modification or composite formation, allowing the wettability of MXene-based membranes to be tailored from superhydrophilic/superoleophobic to amphiphilic states. Additionally, MXenes are highly conductive (*e.g.*, 10 000 S cm^−1^ for Ti_3_C_2_T_*x*_),^35^ allowing not only electro-assisted separation processes but also the application of MXenes in photothermal demulsification strategies. Certain MXene compositions exhibit photothermal conversion efficiencies of >85%, enabling solar-powered interfacial heating, which is sufficient to destabilize emulsions and enhance the separation rate without chemical additives.^36,37^ However, it is important to note that the localized high temperatures generated during photothermal operation, reaching 76–84 °C under one-sun irradiation, may simultaneously accelerate thermally activated oxidative degradation of Ti_3_C_2_T_*x*_ surface sites, particularly during prolonged processing of high-viscosity crude oils. This inherent trade-off between photothermal separation efficiency and oxidative stability remains insufficiently characterized in the literature, and thermal management strategies such as pulsed illumination should be considered in practical system design.^38^

Morphologically, MXenes have a lamellar structure and a high aspect ratio (lateral size of 1–10 µm; thickness of 1–3 nm), leading to a high packing density and extensive interfacial contact when assembled into membranes or sponges.^39,40^ They have the capacity to withstand stresses in filtration cycles as they are mechanically sound when assembled.^41^ Moreover, anti-fouling is facilitated by their negative surface charge (zeta potential between −30 and −50 mV in aqueous media); however, the charge of oil droplets is not intrinsic and depends strongly on the system chemistry, including surfactant type, pH, and ionic strength.^42^ For instance, anionic surfactants (*e.g.*, sodium dodecyl sulfate) impart negative charge to oil droplets.^43^ Therefore, interactions between MXene surfaces and oil droplets should be interpreted within the framework of colloidal interaction theory (*e.g.*, DLVO theory), where the balance between electrostatic repulsion and van der Waals attraction governs droplet adhesion, fouling behavior, and separation efficiency. Another important advantage of MXenes is their chemical and thermal stability in aqueous and organic media. The most commonly studied MXene, Ti_3_C_2_T_*x*_, has been demonstrated to be stable across a pH range of 3–10 and at temperatures up to 300°C under ambient temperatures.^44^ However, a more detailed mechanistic analysis of surface functional group behavior across this pH range is essential for understanding the wettability of MXenes under industrial wastewater conditions. The surface terminations of Ti_3_C_2_T_*x*_, predominantly –OH and –O groups, undergo protonation and deprotonation transitions that fundamentally govern surface charge, hydration layer stability, and oil repulsion capacity.^45^ Under acidic conditions approaching pH 3, surface –OH groups become progressively protonated toward their conjugate acid form, reducing the density of negatively charged surface sites and driving the zeta potential toward neutrality.^46^ This charge neutralization weakens electrostatic repulsion between the MXene surface and oil droplets, destabilizes the structured hydration layer responsible for underwater oleophobicity, and increases the probability of oil adhesion and irreversible fouling. In strongly acidic industrial effluents such as acid mine drainage or pickling liquors operating below pH 3, this protonation effect is expected to be severely compromise oil rejection performance, although the structural integrity of the MXene lattice may be nominally preserved.^38,44^ Conversely, in an alkaline environment above pH 9, surface –OH groups undergo deprotonation to –O^−^ species, maximizing the negative surface charge density and theoretically enhancing electrostatic oil repulsion.^47,48^ However, this enhanced charge also accelerates nucleophilic attack on Ti–C bonds by hydroxide ions, initiating hydrolytic degradation that progressively converts Ti_3_C_2_T_*x*_ to TiO_2_ and releases carbon-containing species into the effluent stream. In strongly alkaline industrial wastewaters such as textile dyeing effluents or caustic cleaning streams operating above pH 12, this hydrolytic mechanism dominates over any wettability benefit conferred by increased surface charge, resulting in rapid structural deterioration and performance collapse.^38^ Between these extremes, in the pH 4–9 window, –OH and –O terminations coexist in a deprotonated state that maintains the maximum negative zeta potential, stable hydration layer integrity, and optimal underwater oleophobicity, defining the practical operational window for pure MXene membranes in industrial applications. These protonation and deprotonation transitions establish that the reported pH 3–10 stability range reflects structural rather than functional stability,^49,50^ and that wettability-dependent separation performance degrades significantly at the boundaries of this range even before structural failure occurs.^51^ This distinction has critical implications for MXene deployment in industrial effluent treatment, where pH environments beyond the optimal window are common and must be managed through composite design or pH pre-adjustment.

**Fig. 1:**
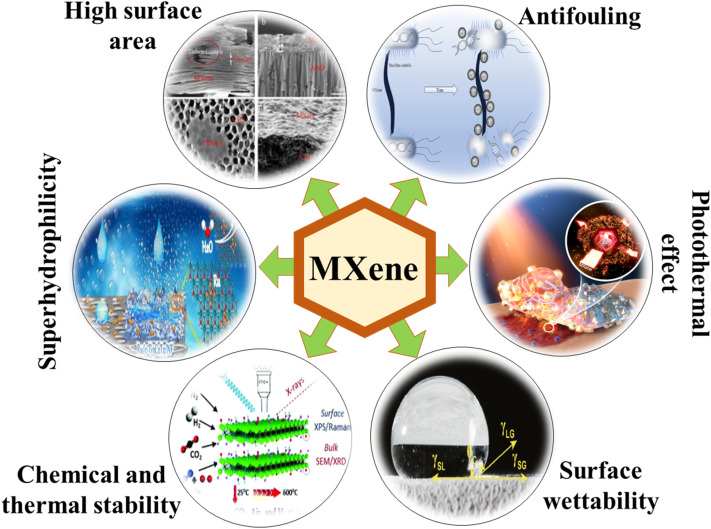
Various properties of MXenes for oil-water separation.

Owing to their high operational stability, MXene-based composites are well suited for operation in strong industrial effluents containing acids, bases, and surfactants. Furthermore, MXenes have a large specific surface area, which ensures a large number of active sites for contact with oil droplets and surfactants, making them ideal building blocks to create hierarchical assemblies, including aerogels, membranes, and sponges with tunable porosity and hierarchical wettability.^52,53^ However, it is important to note that reported surface area values vary considerably across MXene preparations, from below 10 m^2^ g^−1^ for densely restacked laminates to above 100 m^2^ g^−1^ for delaminated or intercalated architectures, reflecting the strong dependence of accessible surface area on synthesis and assembly conditions rather than intrinsic material properties.^54^ All these properties together endow MXenes with unique capabilities as separation materials for immiscible oil-water mixtures and multi-dispersions.^55^ Synergies among these properties have been observed upon incorporation into composite matrices, enabling additional functions such as self-cleaning, responsive wettability, photothermal self-healing, and multi-cycle recyclability (Fig. 1 and Table 1).

**Table 1:** Summary of various oil–water separation mechanisms

Feature	Superhydrophilic/superoleophobic membranes (underwater)	Superhydrophobic/superoleophilic membranes (air)	Adsorption-based and photothermal-assisted separation
Condition	Underwater	In air	Either underwater or under solar illumination
Surface wettability	Hydrophilic to water, repels oil (underwater oleophobic)	Repels water, attracts oil (oleophilic in air)	Adsorbs oil or water depending on surface chemistry; photothermal effect enables surface heating
Driving force	Capillary force, wettability gradient	Gravity or capillary-driven flow through porous materials	Capillarity for adsorption; thermal energy for oil evaporation/separation
MXene role	Provides hydrophilic functional groups and underwater oleophobicity	Creates hierarchical roughness and hydrophobic surface layer	Offers large surface area for adsorption and strong photothermal conversion
Advantages	High separation efficiency, anti-oil fouling	Fast oil uptake, efficient in oily environments	Versatile, high absorption capacity, light-driven separation possible
Challenges	Requires precise surface chemistry, limited to submerged use	Prone to fouling and deterioration in submerged conditions	Reusability, regeneration, and thermal management
Example materials	MXene-coated PAN membranes, GO/MXene hybrids	Salinized MXene on mesh, fluorinated composites	MXene-based aerogels, sponges, or membranes with solar-responsive components
Reusability	High with antifouling design	Moderate; depends on fouling and recovery	Moderate to high; often enhanced by photothermal self-cleaning
Typical application	Separation of oil droplets from underwater emulsions	Skimming oil from water surfaces	Spill recovery, wastewater remediation, and solar-driven separation

### 1.2 Limitations of pure MXene membranes and the necessity of composite design

Despite the exceptional intrinsic properties of MXene nanosheets outlined above, pure MXene membranes face several fundamental limitations that render them practically unsuitable for direct industrial deployment in oil–water separation, collectively motivating the composite material strategies discussed in Section 2. The most critical limitation is oxidative instability in aquatic environments. Ti_3_C_2_T_*x*_ nanosheets are thermodynamically susceptible to oxidation when exposed to dissolved oxygen and water, progressively converting surface carbide sites to TiO_2_ and disrupting the structural integrity of lamellar membranes. This oxidative degradation is particularly severe at elevated temperatures and under acidic or strongly alkaline conditions, leading to irreversible flux decline and loss of selective transport properties over operational timescales relevant to industrial application.^38,44^ The second critical limitation is interlayer restacking and nanochannel collapse. During membrane fabrication *via* vacuum filtration or evaporation-induced assembly, the strong interlayer van der Waals attractions between MXene nanosheets drive spontaneous restacking into densely packed laminates with nonuniform sub-nanometer channels. This restacking severely reduces effective porosity, dramatically attenuates water flux, and creates structural heterogeneity that compromises separation selectivity.^56^ In practical terms, restacking transforms the theoretically open nanochannel network of individual MXene sheets into a largely impermeable dense film, negating the permeability advantages that make MXenes attractive. Third, pure MXene membranes exhibit poor mechanical robustness under the cyclic hydraulic stresses of filtration operation. Freestanding MXene laminates are brittle and prone to cracking or delamination under transmembrane pressure, particularly when interlayer cohesion is weakened by hydration swelling. This mechanical fragility limits operational pressure ranges and shortens membrane lifespan under realistic processing conditions.^41^ Fourth, the high surface energy and negative surface charge of pure MXene membranes, while beneficial for oil repulsion, also render them susceptible to irreversible fouling by cationic surfactants and charged organic contaminants present in real industrial effluents, which can neutralize surface charge and collapse the hydration layer responsible for underwater oleophobicity.^42^ Collectively, these four limitations, oxidative instability, interlayer restacking, mechanical fragility, and charge-dependent fouling susceptibility, establish that pure MXene membranes cannot yet meet the durability, flux stability, and operational resilience requirements of industrial oil–water separation. These limitations define the design space that composite engineering strategies must address, providing the logical foundation for the composite material classifications presented in Section 2.

## 2. Types of MXene-based composites

### 2.1 MXene–polymer composites

The composites of MXenes with polymer are promising materials in the field of advanced oil-water separation due to the excellent surface chemistry and electrical conductivity of MXenes combined with the flexibility, processability, and mechanical strength of polymeric scaffolds. The incorporation of MXene nanosheets into polymers, including polyvinylidene fluoride (PVDF) and polyacrylonitrile (PAN), has facilitated the fabrication of hydrophilic, selectively wetted or anti-fouling membranes.^57,58^ These composites can be prepared using various techniques such as physical blending, surface coating, and layer-by-layer assembly, each offering distinct advantages in terms of membrane structure and performance. The obtained hybrid membranes are characterized by high separation efficiency, high mechanical stability, and reusability, making them desirable for industrial-scale oily wastewater treatment and prolonged filtration use.^42^ The versatile functionality of MXenes also endows these membranes with additional features such as antibacterial activity and photothermal responsiveness, further expanding their applicability under dynamic environmental conditions.^59^ The rigid 2D MXene flakes strengthen the polymer and impart high –OH/-O terminations, making it more hydrophilic.^60^ For example, a PVDF mixed-matrix membrane with 0.5 wt% Ti_3_C_2_T_*x*_ MXene showed a dramatically lower water contact angle (38°) than PVDF, along with a pure water flux of 538 L m^−2^ h^−1^ and nearly 100% rejection of a model dye. This membrane also exhibited excellent anti-fouling (flux recovery of 85.6%).^61^ Similarly, an optimized 0.2 wt% MXene/PVDF membrane achieved a huge water permeance (1242 kg m^−2^ h^−1^ bar^−1^) and 99% oil rejection over multiple cycles.^62^ These results illustrate that MXene/polymer composites combine improved mechanical integrity (from the strong MXene network) with ultrafiltration selectivity (due to narrow nanochannels and surface charges). MXene–polymer composites are prepared by either blending MXenes into a polymer casting solution or coating/laminating MXene layers onto a polymer support. In the blend approach, MXene nanosheets are dispersed in the polymer dope before membrane casting. For instance, one study cast a PVDF/MXene mixed-matrix membrane by bulk modification (mixing MXene with a PVDF solution), which significantly improved the fouling resistance compared to pure PVDF.^61^ Alternatively, MXenes can be layered on polymer films (*e.g.* vacuum filtration of MXene onto PVDF) to create thin surface coatings. Both methods yield robust membranes: blending ensures MXene–polymer interlocking (enhancing strength), while coating allows control of the MXene layer thickness and surface chemistry. The use of MXene significantly improves anti-fouling. The smooth, hydroxyl group-rich MXene surface prevents pollutant adhesion and, in most cases, enables only reversible binding.^63^ MXene-based composite UF membranes exhibit significantly higher fouling flux-recovery ratios (FRR) than pure polymers, as demonstrated in previous studies.^61,62^ In another study, incorporating MXene into a PSF (polysulfone) membrane improved BSA rejection to over 90% (compared to 77% with pure PSF) and provided an FRR of 76%.^64^ These advancements demonstrate that MXene–polymer composites not only exhibit enhanced filtration performance but are also more readily regenerated, thus enabling their reuse.

### 2.2 MXene–carbon/nanomaterial hybrids

**Fig. 2:**
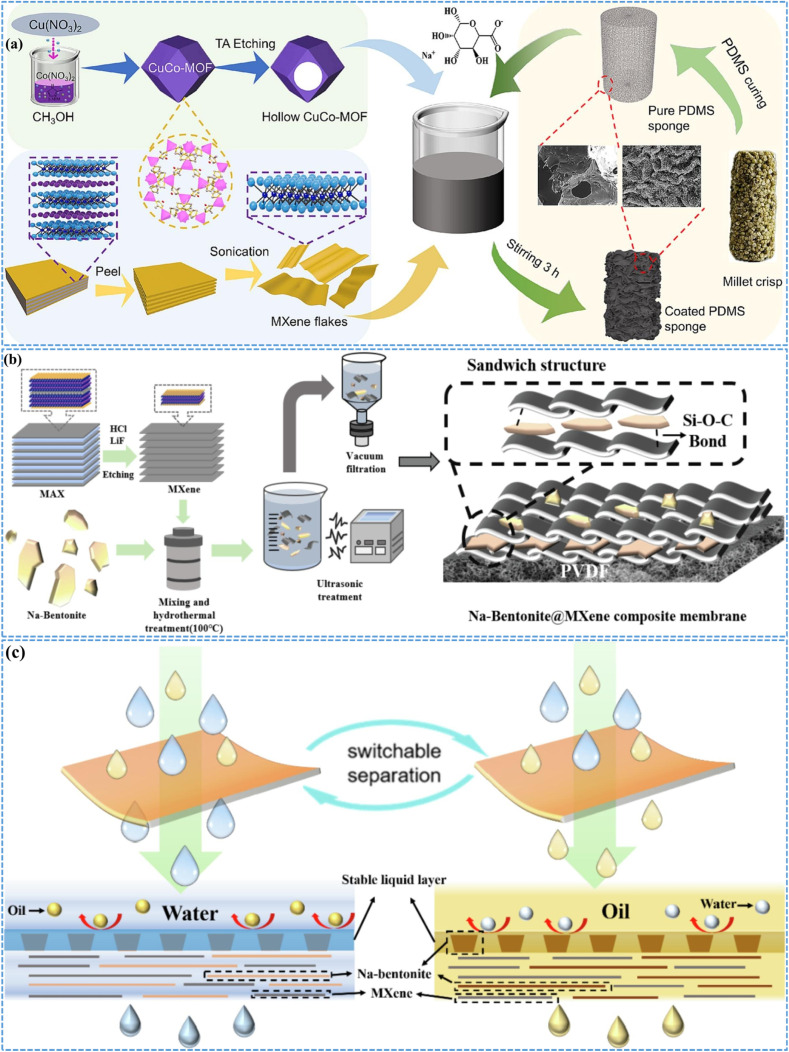
(a) Schematic of the stepwise fabrication of the MXene/H–CuCo-MOF@PDMS sponge. Reproduced with permission from ref. 74. Copyright 2025, Elsevier. (b) Schematic depicting the structural assembly of the NBM composite membrane and (c) mechanistic schematic elucidating the switchable wettability behavior governing oil/water emulsion separation. Reproduced with permission from ref. 83. Copyright 2023, Elsevier.

The rational design of MXene-based composites with carbonaceous and nanostructured materials, including carbon nanotubes (CNTs), graphene oxide (GO), and MOFs, has garnered considerable interest in the development of oil-water separation technologies. These hybrid materials synergistically integrate the physicochemical strengths of their constituents: MXenes possess rich surface functional groups, high conductivity, and high hydrophilicity; CNTs exhibit mechanical robustness and flexibility; GO possesses a high surface area and defect-rich architecture; and MOFs feature porous structures and chemical tunability.^65–68^ Thus, the combination of these materials results in hierarchical architectures and interconnected porous channels, yielding membranes with enhanced permeability, selectivity and anti-fouling performance.^69^ These nanohybrid membranes are generally prepared *via* vacuum-assisted self-assembly, layer-by-layer deposition or *in situ* growth to provide scalability and structural control.^70^ The resulting membranes not only exhibit high separation performance, but also long-term durability and high recyclability, making them ideal materials for wastewater treatment, oil spill cleanup, and remediation of industrial oily effluents. The hybridization approach opens a novel paradigm in creating multifunctional and high-efficiency MXene-based materials for use in sustainable environmental applications. MXene was used in conjunction with carbon nanomaterials to create high porosity 3D network membranes with expanded channels and interconnected pores.^71^ For instance, the incorporation of multi-walled carbon nanotubes (MWCNTs) into a Ti_3_C_2_T_*x*_ MXene laminate leads to an increase in the interlayer spacing and water permeability. The membranes with a higher MWCNT content showed dramatically higher water flux than that of the pure MWCNT membrane, reaching 80 L m^−2^ h^−1^ due to their expanded nanochannels. Notably, even the addition of a small amount of MXene (1 mg in the composite) significantly boosted the oil–water separation performance: the permeate flux and oil rejection efficiency both increased in the presence of MXene.^2^ MXene–carbon hybrids merge the advantages of both materials: the conductivity and flexibility of CNT/graphene and the hydrophilicity and tunable chemistry of MXene, yielding membranes with superior porosity, flux and separation efficiency. Integrating MOFs with MXenes yields 2D/2D hybrids that combine the high conductivity and hydrophilicity of MXenes with the crystalline structure and porosity of MOFs.^72^ MOFs can be grown *in situ* on MXene sheets or mixed layer-by-layer, yielding membranes with hierarchical pores (micropores from the MOF and nanochannels from MXene).^73^ These MXene/MOF composites often show enhanced adsorption capacity and photocatalytic activity, making them useful for contaminant degradation and ion sieving. For example, a hydrophobic MXene/H–CuCo-MOF@PDMS sponge was synthesized, as shown in Fig. 2a, where MXene and hollow CuCo-MOF create efficient electron–transport pathways and abundant adsorption sites. Stress mismatch between MXene and PDMS forms a wrinkled structure that prevents MXene stacking and enhances photothermal performance. Rapid sponge heating to 80 °C under one-sun irradiation allows rapid uptake of viscous oils. It achieves high separation efficiencies (95% in diesel and rapeseed oil, 82.7% in crude oil), along with enhanced durability, mechanical recovery, and salt resistance.^74^

### 2.3 MXene–metal oxide/chitosan/clay composites

Surface functionalization of MXenes with inorganic and bio-based materials such as metal oxides, chitosan, and clay has become a potential approach for the development of multifunctional membranes with improved oil-water separation capabilities. The interfacial chemistry and structural diversity of additives are used in these composite systems to tune surface wettability, enhance chemical and thermal stability, and add new functionality. Metal oxides (TiO_2_, ZnO, and Fe_3_O_4_) confer oxidative resistance, catalytic activity and roughness-enhanced wetting behavior, while chitosan, a biodegradable polysaccharide, provides hydrophilicity, antimicrobial activity and mechanical flexibility.^75–77^ Meanwhile, layered clays, including montmorillonite, provide excellent cation-exchange capacity, thermal stability and micro- and nanoporous structures, yielding selective permeation and interfacial properties.^78^ These properties are advantageous in the separation of stabilized emulsions or viscous oil systems. Additionally, the photothermal conversion potential of MXene due to its strong near infrared absorption and localized surface plasmon resonance has been successfully utilized in solar-assisted oil-water separation.^79^ Upon exposure to solar irradiation, the MXene-based composite membrane generates localized heating, which reduces the viscosity of oil pollutants and interfacial adhesion while enabling permeation-driven separation without the need for external power input.^80^ Composites of MXene with metal oxides (*e.g.*, TiO_2_ and ZnO), biopolymers (chitosan) or clay minerals can precisely regulate the wettability and stability of membranes.^81^ For instance, an MXene/ChNC@TiO_2_ membrane was fabricated by loading TiO_2_-coated chitin nanocrystals between MXene layers, thereby forming hydrophilic water channels that greatly increased water transport. When separating micrometer-size emulsions (oil droplets 5–25 µm), the membrane achieved an oil rejection rate of over 99%, which was significantly higher than the 81–89% rejection rate obtained with the pure MXene membrane. The composite membrane also exhibited higher emulsion permeance, which increased from 580–620 to 740–816 L m^−2^ h^−1^ bar^−1^.^82^ In another study, Na-bentonite was embedded into MXene nanosheets to form Na-bentonite@MXene composite membranes *via* hydrothermal pretreatment and vacuum self-assembly, as shown in Fig. 2b. The membranes showed strong oil/water separation performance with a 96% rejection rate and 86% flux recovery after eight cycles. The MXene–bentonite sandwich structure provided stability and created a micro–nano-structured surface, enabling switchable wettability for different oil–water mixtures (Fig. 2c). This design offers effective and durable MXene-based membranes for oily wastewater treatment.^83^

## 3. Oil-water separation *via* various systems

**Fig. 3:**
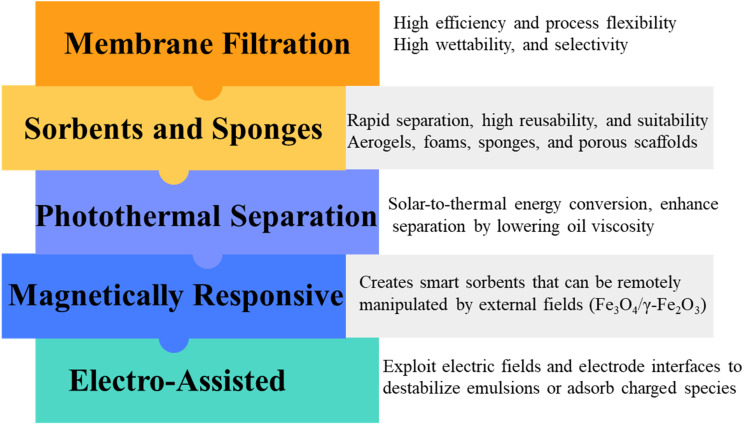
Various oil–water separation systems and their respective advantages.

This section systematically discusses the various MXene-enabled oil–water separation systems (Fig. 3), emphasizing how material properties translate into distinct operational mechanisms across different configurations. The relationship among system design, separation efficiency, and governing physicochemical principles is critically examined.

### 3.1 Membrane filtration

Membrane filtration represents one of the most promising and practically scalable technologies for oil–water separation, offering high efficiency, modularity, and process flexibility.^16^ Cross-flow and dead-end filtration systems are the most commonly used types of membrane set-ups, each with unique advantages based on the separation and the scale of operation.^84^ Over the past years, membrane matrices based on MXene nanocomposites have been incorporated to produce next generation membranes with desired wettability, enhanced mechanical integrity, and adjustable selectivity to four-phase oil or water phases. These superior membranes could be tailored to promote either a hydrophilic or oleophilic behavior, which could be used in a wide range of oily wastewater streams, such as emulsified systems.^85,86^ The scalability, tunability, and adaptability of membrane systems make them an attractive platform for real-world wastewater remediation applications. The following section highlights reported studies utilizing MXene-based membranes in various filtration modes for enhanced oil–water separation performance. Two-dimensional Ti_3_C_2_T_*x*_ MXene nanosheets possess hydrophilic surface terminations and lamellar channels whose properties can be tuned by interlayer chemistry, resulting in underwater super-oleophobicity, rapid water transport, and anti-fouling potential for oil–water separation.^87^

It is important to distinguish between two fundamentally different separation mechanisms operating in MXene-based membranes. Interlayer nanochannels, typically on the order of 1–2 nm, primarily regulate the transport of water molecules, hydrated ions, and small solutes through size-sieving and confinement effects.^41,88^ However, emulsified oil droplets, which generally range from nanometers to several micrometers, are significantly larger than these nanochannels and therefore cannot be rejected *via* direct steric exclusion.^89–91^ Instead, oil rejection is predominantly governed by interfacial wettability. The hydrophilic MXene surface forms a stable hydration layer that generates an energetic barrier against oil adhesion and penetration.^92^ Consequently, while nanochannels control permeation flux and selectivity for small species, wettability and interfacial energy dominate the rejection of oil droplets, particularly in emulsified systems. The cross-flow geometry sweeps foulants away from the membrane surface, whereas the dead-end geometry promotes foulant accumulation at the surface. Accordingly, MXene laminates and MXene-polymer nanocomposites have been engineered for use in both configurations to reject free oil and nanoemulsions at low transmembrane pressures.^93^

**Fig. 4:**
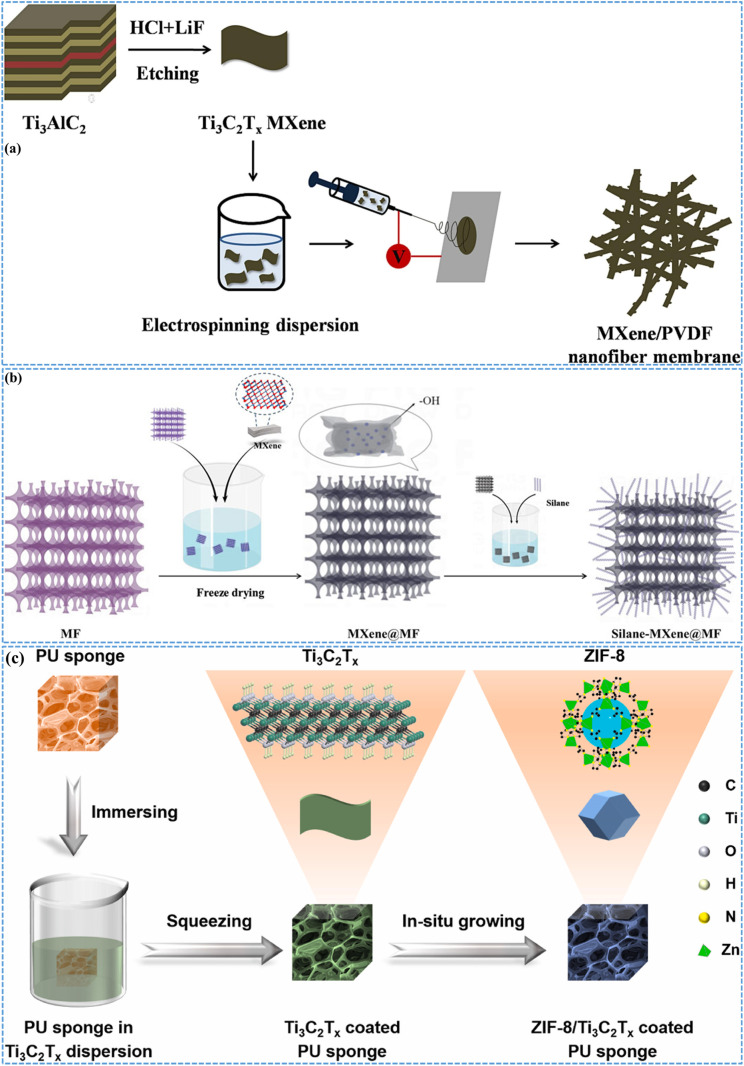
(a) Schematic of the fabrication process of the MXene/PVDF nanofibrous membrane. Reproduced with permission from ref. 95. Copyright 2024, Elsevier. (b) Schematic illustrating the preparation and surface functionalization of the silane-MXene@MF composite. Reproduced from Frontiers from ref. 117, which is open access and permits unrestricted use of materials under the terms of the Creative Commons CC-BY. (c) Schematic of the synthesis route for the PU–Ti_3_C_2_T_*x*_–MOF sponge. Reproduced with permission from ref. 118. Copyright 2023, Elsevier.

For instance, Saththasivam *et al.*^94^ blade-coated MXene ink onto low-cost cellulose paper to create a flexible, freestanding membrane. This membrane achieved >99% rejection of diverse oil-in-water emulsions with a water flux >450 L m^−2^ h^−1^ bar^−1^, and exhibited simple, chemical-free fouling recovery *via* physical rinsing. The authors attributed the performance to hydrophilic surface properties and tortuous transport pathways, in which wettability-controlled interfacial rejection governs oil separation, while nanochannels primarily facilitate selective water transport. Moreover, durability under bending and repeated cycles demonstrates the low-cost scalability of modular dead-end cartridges. In another study, Zhang *et al.* assembled Na-bentonite@MXene laminates with pH- and ion-responsive channels to switch between water-selective and oil-selective states. The surfaces toggled between superhydrophilic/underwater super-oleophobic and superhydrophobic/oleophilic, enabling the separation of both oil-in-water and water-in-oil emulsions within the same module. Interlayered clay improved mechanical integrity and restricted swelling, stabilizing the nanochannels under operation. This work suggests bidirectional MXene membranes for plants that process variable oily waste streams.^83^ In another study, MXene (Ti_3_C_2_T_*x*_)-embedded PVDF nanofiber membranes were fabricated *via* electrospinning to create highly hydrophobic, photothermal separation materials (Fig. 4a). The membranes delivered exceptionally high oil fluxes, including 16 977.9 L m^−2^ h^−1^ for hexane and 1018.7 L m^−2^ h^−1^ for heavy mineral oil under 1-sun-assisted vacuum filtration. They achieved over 99% rejection of heavy mineral oil/saltwater emulsions while maintaining stability in acidic, alkaline, and high-UV environments. These results demonstrate the strong potential of these membranes for efficient oil–water separation, particularly in challenging marine spill conditions.^95^ Similarly, Amari *et al.* fabricated a WO_3_/MXene-PAES composite membrane having superhydrophilic and superoleophobic properties to allow oily wastewater to be treated effectively. The optimized pore structure and enhanced wettability resulted in a high separation efficiency and water flux of 99.98% and 6.4 L m^−2^ h^−1^ bar^−1^, respectively. Its high fouling resistance and fast recovery time of 0.25 h also ensured repeatable and stable oil and water separation. Overall, synergistic WO_3_/MXene modification resulted in a strong membrane that could be used in high-performance and self-cleaning oil water purification.^96^ A critical comparison of flux values reported across MXene-based membrane studies reveals that discrepancies spanning several orders of magnitude, ranging from 6.4 L m^−2^ h^−1^ for WO_3_/MXene composite membranes^95^ to 16 977.9 L m^−2^ h^−1^ for photothermal MXene/PVDF nanofiber membranes,^94^ do not indicate contradictory material performance but rather reflect fundamentally different operating conditions, oil types, emulsion droplet size distributions, and driving forces. Specifically, the ultra-high flux values reported for solar-assisted nanofiber membranes are achieved under vacuum-assisted filtration using low-viscosity light oils such as hexane, whereas lower flux values correspond to gravity-driven or pressure-limited systems processing viscous or heavy oils. Furthermore, emulsion droplet size critically governs rejection efficiency: membranes tested against micrometer-scale droplets inherently report higher flux than those challenged with nanoemulsions requiring tighter nanochannel confinement. This contextual reconciliation underscores the necessity of standardizing reporting conditions, including driving pressure, oil viscosity, droplet size distribution, and temperature, to enable meaningful cross-study comparison and rational membrane design. An effective prefilter route is coated with meshes to attain superhydrophilicity and underwater super-oleophobicity, which enables de-oiling by gravity, followed by fine MXene filtration. Yuan *et al.* reported fabrication routes (spray/spin coating and thermal treatment) to produce meshes with high flux and strong anti-oil adhesion, and these processes reduce particulate/oil loading on MXene. These meshes demonstrate that hierarchical wettability can be combined with MXene membranes to extend operational lifetime. The mesh design is inherently scalable and can be retrofitted into cross-flow lines.^97^ A key limitation is MXene swelling and delamination in water, which destabilize selectivity, while robust intercalation or crosslinking (*e.g.*, sodium-alginate or clay) fixes gallery spacing and resists hydration. Oxidative decomposition of Ti_3_C_2_T_*x*_ at high potentials or during storage may cause performance degradation; to prevent this, antioxidants can be preserved through surface passivation or polymer encapsulation. Viscous oils contribute to flux depletion, and introducing photothermal or photocatalytic (*e.g.*, WO_3_/MXene or MXene-MWCNT) functionalities into the MXene layer enables *in situ* light-assisted cleaning and viscosity and enhances flux recovery in cyclic processes. Finally, in high-TDS feed cases, MXene membranes should be used with superwetting pre-meshes or coalescers to eliminate oil/solid loads and increase the cleaning duration. However, a critical limitation of the current literature is that the majority of reported separation studies employ idealized laboratory-simulated oil-water mixtures. Real industrial effluents, including petrochemical wastewater, offshore produced water, and food-processing discharge, present substantially more complex compositional challenges that remain largely unevaluated for MXene-based systems.^98^ Specifically, three environmentally realistic conditions require urgent attention. First, complex surfactant mixtures common in industrial effluents contain both anionic and cationic species simultaneously,^99^ and cationic surfactants are known to adsorb onto the negatively charged MXene surface, neutralizing surface charge, collapsing the hydration layer responsible for underwater oleophobicity,^100–102^ and significantly reducing oil rejection efficiency. Second, high salinity environments such as offshore produced water with total dissolved solids exceeding 30 000 mg L^−1^ compress the electrical double layer on MXene surfaces through ionic screening,^103^ reducing electrostatic oil repulsion and promoting colloidal destabilization of emulsions in ways that may simultaneously enhance or impair separation depending on droplet charge state. Third, variable temperature operation, particularly in cold marine environments below 10 °C or hot industrial discharges above 60 °C, affects both oil viscosity and MXene surface chemistry in ways that current laboratory evaluations at ambient temperature do not capture.^104–106^ The absence of systematic performance data under these realistic conditions represents a significant gap between laboratory demonstrations and industrial deployment readiness, and future studies must prioritize the evaluation of MXene-based separation systems against standardized complex feed matrices rather than idealized model emulsions.

A critical examination of reported flux data across pH environments reveals notable inconsistencies that warrant mechanistic interpretation. Several studies report a monotonic increase in water flux under alkaline conditions for Ti_3_C_2_T_*x*_-based membranes, attributable to progressive deprotonation of surface –OH groups, which enhances surface charge density and electrostatic repulsion of oil droplets, while simultaneously widening effective nanochannel spacing through interlayer electrostatic expansion.^83,87^ However, conflicting reports demonstrate flux deterioration above pH 9, linked to accelerated oxidative degradation of MXene sheets under alkaline conditions, converting Ti_3_C_2_T_*x*_ to TiO_2_ and collapsing the lamellar structure.^38^ Under acidic conditions (pH < 4), protonation of surface terminations reduces the negative zeta potential toward neutrality, weakening electrostatic oil repulsion and promoting fouling-induced flux decline.^42^ These contradictions are not irreconcilable; rather, they reflect differences in membrane fabrication strategies, intercalant chemistry, and MXene oxidation state at the time of testing. Membranes stabilized by crosslinking agents such as sodium alginate or bentonite intercalation demonstrate pH-stable flux across pH 3–10,^83^ whereas freestanding MXene laminates show strong pH sensitivity. This distinction suggests that flux-pH behavior is governed not by MXene surface chemistry alone, but by the coupled interactions among interlayer spacing stability, surface termination protonation state, and structural integrity under aqueous conditions.

Based on collective evidence, we propose a three-regime framework for pH-dependent flux behavior in MXene membranes: (i) acidic regime (pH < 4): protonation of –OH/–O terminations reduces surface charge, contracts the electrostatic double layer, decreases interlayer repulsion, and narrows effective nanochannel apertures, resulting in reduced flux and increased oil-adhesion probability; (ii) neutral-to-mildly alkaline regime (pH 4–8): terminations remain deprotonated, zeta potential is maximally negative (−30 to −50 mV), interlayer spacing is stable, and flux is optimized; and (iii) strongly alkaline regime (pH > 9): surface oxidation accelerates, structural delamination occurs, and paradoxically, flux may transiently increase due to structural loosening before declining irreversibly due to channel collapse. This three-regime framework reconciles the apparent contradictions in reported data and provides a predictive basis for designing pH-resilient MXene membranes targeting industrial effluents with variable pH profiles.

### 3.2 Sorbents and sponges

Oil water separation systems *via* adsorption have attracted considerable attention due to their simple operation, high separation kinetics, high reusability and applicability to both emulsified and free oil phases. Structured materials are normally used in these systems including aerogels, foams, sponges, and porous scaffolds, which are commonly functionalized with nanomaterials to achieve desired wettability profiles and high sorption capacity.^107^ In this regard, MXene-based composites have emerged as outstanding candidates towards adsorption-based platforms due to their high surface area, controllable surface functionalities, and structural integrity. MXene-modified sponges and aerogels are superoleophilic/superhydrophobic or superhydrophilic/underwater when hybridized with carbonaceous, polymeric, or inorganic structures to allow the selective adsorption and recovery of oil or water phases under ambient or severe conditions.^55,108^ The adsorption behavior in these systems is governed by capillary-driven transport and interfacial thermodynamics. In porous MXene-based sorbents, oil uptake is primarily governed by capillary pressure,^109^ which can be described by the Young–Laplace equation: Δ*P* = 2*γ* cos *θ*/*r*, where *γ* is the interfacial tension, *θ* is the contact angle, and *r* is the effective pore radius.^110^ For superoleophilic surfaces (*θ* < 90°), the capillary pressure becomes negative, driving spontaneous oil infiltration into the porous network.^111,112^ Conversely, superhydrophobic or underwater superoleophobic configurations introduce an energy barrier that inhibits water penetration. In addition, interfacial energy minimization governs selective absorption, whereby the system preferentially adsorbs the phase that minimizes the total surface free energy.^113^ These mechanisms collectively account for the high selectivity and rapid uptake observed in MXene-based aerogels and sponges. These systems can be tailored to the target pollutant, and due to their multi-cycle regeneration ability, they are especially attractive as a method for sustainable wastewater treatment.^114^ The subsequent subsection presents key studies that demonstrate the utility of MXene-derived adsorbents in oil–water separation applications. When MXene sheets are integrated into lightweight porous frameworks, PU foams, melamine sponges, cellulose- or graphene-reinforced aerogels, and 3D-printed scaffolds, they confer superhydrophobic/oleophilic or underwater super-oleophobic surfaces along with mechanical strength, electrical/thermal response, and high surface area for rapid oil uptake.^115^ Owing to their hierarchical porosity and low density, their absorption capacities typically reach 100–160 times the sorbent weight for oils and organic solvents, with fast squeezing-based regeneration.^116^

For instance, Li *et al.* created a monolithic 3D MXene sponge *via* freeze-casting and surface modification to achieve superhydrophobicity and strong photothermal conversion. Under solar irradiation, localized heating decreased oil viscosity and drove rapid imbibition, and the sponge exhibited rapid separation of oil/water mixtures and high organic-solvent uptake. Reusability was demonstrated over many absorption–squeezing cycles without structural collapse.^116^ Similarly, a multifunctional MXene/AuNP-reinforced aerogel was fabricated *via* directional freeze-drying to enhance crude oil cleanup, especially for highly viscous oils. The hydrophobic aerogel (145°) demonstrated a high absorption capacity of 45.7–85.6 g g^−1^ and maintained strong mechanical resilience with 85.7% strain retention after 30 compression cycles. Due to its effective photothermal conversion, the aerogel was quickly heated to 76 °C, taking only 10 s to absorb viscous crude oil in the presence of light. These outcomes indicate its high capability of quick light-assisted crude oil separation under challenging spill conditions.^28^ Similarly, Wang *et al.* coated silane-modified MXene onto melamine foam (Fig. 4b) and generated a strong superhydrophobic/oleophilic network, which separated immiscible mixtures and emulsions. In diesel/water emulsions, the foam achieved a flux of 1354 L m^−2^ h^−1^ with a separation efficiency of 93% in a gravity-assisted system and selectively absorbed heavy chlorinated oils. The nano-rough MXene domains facilitated coalescence of micron/nano-droplets as well as repelling water. The foam retained its performance after repeated use, supporting its reusability.^117^ Deng *et al.* deposited MXene/MOF hybrid nanocoatings on PU sponges to synergize surface roughness and chemical functionality (Fig. 4c). The resulting superhydrophobic sponges exhibited high oil uptake, rapid separation of immiscible mixtures, and chemical durability, while the MOF component supplied additional adsorption/active sites. Recyclability by squeezing or solvent washing preserved capacity across cycles. The work illustrates that MXene acts as an interfacial glue, anchoring multifunctional porosity to commodity foams.^118^ These observations highlight that separation in MXene-based sorbents is not solely a function of material composition but is fundamentally governed by coupled transport and interfacial phenomena.

A critical comparison of reported absorption capacities across MXene-based sorbents reveals apparent conflicts that require mechanistic resolution. Absorption capacities ranging from 27 g g^−1^ for magnetic PDMS-coated sponges^119^ to 85.6 g g^−1^ for MXene/AuNP aerogels^28^ and exceeding 100 g g^−1^ for graphene-reinforced MXene aerogels^115^ reflect differences in material porosity, density, and oil viscosity rather than intrinsic contradictions. Low-density aerogels with hierarchical macroporosity and superoleophilic surfaces demonstrate higher gravimetric uptake due to their greater void volume available for oil infiltration, driven by capillary pressure described by the Young–Laplace equation. Conversely, denser sponge-based systems offer mechanical robustness and cyclability at the cost of absolute capacity. Furthermore, conflicting reports on reusability, where some studies report stable performance over 15 cycles^120^ while others report significant capacity fade after 5 cycles, are reconciled by differences in regeneration strategy: thermal or photothermal regeneration preserves pore structure more effectively than mechanical squeezing alone, which induces cumulative structural fatigue. These distinctions establish that capacity and reusability metrics are system-specific and must be interpreted within the context of material architecture and regeneration mechanism.

Leaching of nanocoatings into water is a concern; therefore, covalent grafting, MOF/MXene hybridization, or polymer encapsulation can reduce particulate release. Mechanical fatigue under repetitive squeezing can drive capacity fading; in this case, CNF-reinforcement and wood-inspired lamellae preserve structure under cyclic loads. Sorbents are best suited for episodic spills but less suited to continuous low-ppm polishing; therefore, integrating sorbent pre-cleanup with a downstream MXene membrane train offers continuous treatment and solvent recovery. Finally, biofouling/organic deposition can be remediated by leveraging MXene photothermal/Joule heating for on-sorbent self-cleaning.

### 3.3 Photothermal separation

**Fig. 5:**
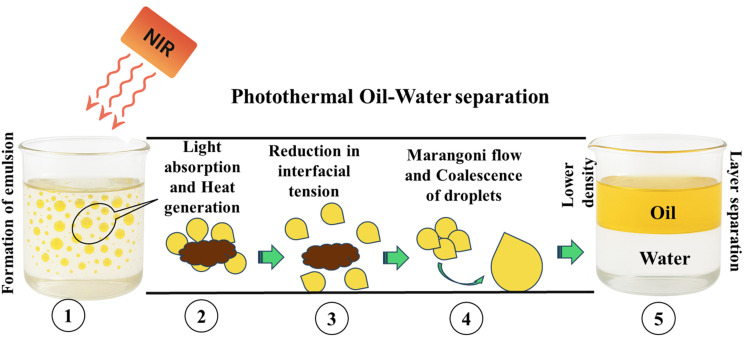
Schematic of photothermal oil–water separation.

Photothermal-assisted oil-water separation systems are an emerging technology that exploit the capability of solar-to-thermal energy conversion to improve separation performance, especially under viscous and emulsified oil conditions. The key to this strategy is the use of photothermally active materials such as MXenes, particularly Ti_3_C_2_T_*x*_, which have demonstrated incredible potential due to their strong light absorption, rapid photoresponse, and high thermal conductivity.^121^ MXene-based materials can rapidly generate localized heat upon light irradiation, thereby reducing the viscosity of oil, destabilizing emulsions, and enhancing interfacial transport dynamics (Fig. 5). The enhancement in separation performance under photothermal conditions is fundamentally linked to the temperature dependence of oil viscosity and interfacial properties. The viscosity of most hydrocarbon oils decreases exponentially with increasing temperature, which significantly improves mass transport and capillary flow within porous structures.^122,123^ This viscosity–temperature relationship reduces flow resistance and facilitates faster permeation or absorption of viscous oils. Additionally, localized heating weakens interfacial adhesion between oil droplets and solid surfaces, lowering the energy barrier for detachment and mitigating fouling.^124^ From a transport perspective, photothermal effects therefore act by simultaneously enhancing fluid mobility and reducing interfacial resistance, rather than altering intrinsic membrane selectivity. The thermally induced separation process, when incorporated into smart membranes or porous scaffolds, allows manipulation of the oil and water phases energy efficiently and without contact, even under natural sunlight.^125,126^

**Fig. 6:**
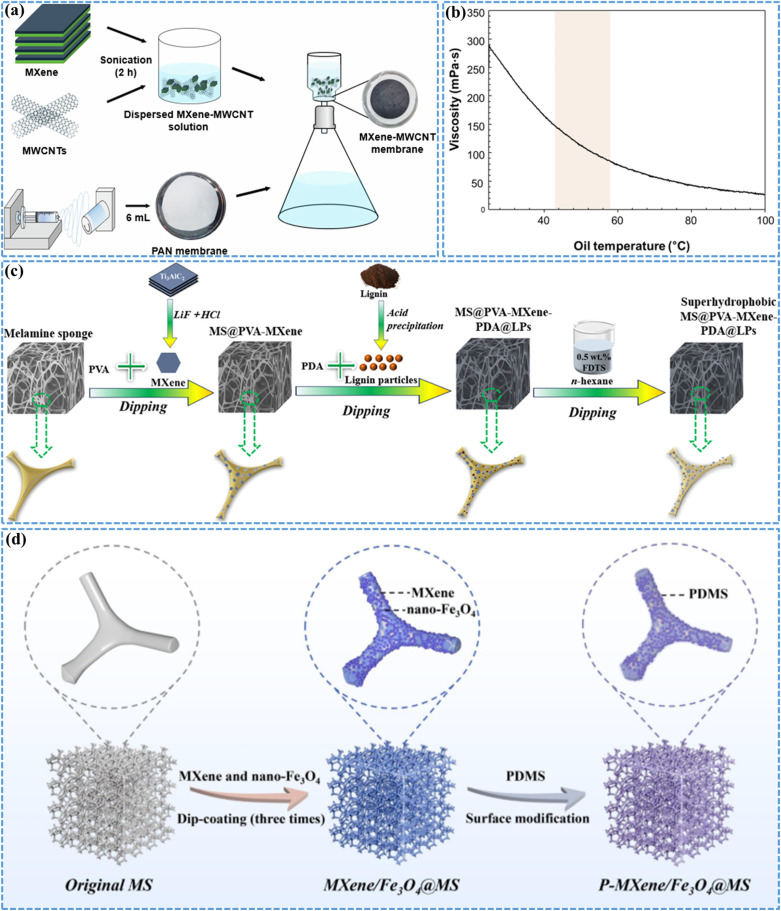
(a) Schematic outlining the membrane fabrication process and (b) variation in oil viscosity as a function of temperature for different oil types. Reproduced with permission from Nature from ref. 2, which is open access and permits unrestricted use of materials under the terms of the Creative Commons CC-BY. (c) Schematic of the stepwise preparation of the MS@PVA–MXene–PDA@LPs composite. Reproduced with permission from the American Chemical Society from ref. 119. (d) Schematic illustrating the construction pathway of the P-MXene/Fe_3_O_4_@MS architecture. Reproduced from John Wiley and Sons ref. 131, which is open access and permits unrestricted use of materials under the terms of the Creative Commons CC-BY.

This section will explore the studies that explain how MXene-enabled photothermal platforms are reshaping the paradigm of oil-water remediation based on solar energy. MXenes have broadband solar absorption and high photothermal conversion efficiencies, which enable *in situ* heating that increases viscous oil resistance, facilitates interfacial dewetting and removes fouling in membranes and sorbents.^127^ Sunlight-driven operation can lower the energy cost of de-oiling, especially for heavy oils and stabilized emulsions, by locally thinning boundary layers and desorbing foulants without chemical cleaning.^128^ Pairing MXenes with conductive carbons or photocatalysts further strengthens light harvesting and thermal management for sustainable separations. For instance, Byun *et al.* embedded multi-walled carbon nanotubes in MXene to form a heatable separation membrane (Fig. 6a), enabling on-demand photothermal cleaning. Under illumination, the membrane achieved a 60% flux recovery after fouling while maintaining 99% oil rejection over four cycles, compared with only 7% recovery without photothermal assistance under identical conditions. Local heating lowered oil viscosity and weakened foulant adhesion, as shown in Fig. 6b, thereby reducing cleaning time and water consumption. This approach demonstrates improved practical reusability of membranes for industrial applications.^2^ Comparing photothermal performance across MXene-based systems reveals an important mechanistic distinction that is frequently overlooked in the literature. Studies reporting temperature rises to 76 °C under NIR irradiation for MXene/AuNP aerogels,^28^*versus* 57.5 °C for MXene/lignin sponges^129^ and 64 °C for Fe_3_O_4_/MXene/lignin composites,^130^ reflect differences in photothermal conversion pathways rather than intrinsic material quality. Gold nanoparticles contribute localized surface plasmon resonance absorption in the visible range, synergizing with the broadband NIR absorption of MXenes to generate higher local temperatures. Lignin particles, by contrast, contribute primarily through π–π stacking-enhanced light scattering rather than plasmonic mechanisms. This mechanistic distinction has direct practical implications: plasmonic-enhanced systems are more effective for highly viscous crude oils requiring greater viscosity reduction, while lignin-based systems offer cost and sustainability advantages for lighter oil applications. A unified photothermal performance metric incorporating conversion efficiency, temperature rise rate, and oil viscosity reduction factor would enable more meaningful cross-system comparison and guide material selection for specific spill remediation scenarios.

Wang *et al.* prepared melamine sponges decorated with MXene and lignin particles to couple robust superhydrophobicity with strong photothermal response, as shown in Fig. 6c. Under simulated/solar light, the sponge rapidly heated and accelerated the capture and release of viscous oils, enabling continuous separation with low external energy input. Moreover, it separates oil-water mixtures and recovers viscous crude oil *via* sunlight-induced photothermal heating, reaching 57.5 °C and thereby reducing oil viscosity. The material adsorbs up to 91.6 times its weight, achieves a 99.4% separation efficiency, and maintains anti-icing performance after 50 cycles, offering a simple, low-cost strategy for efficient oil cleanup and active deicing.^119^ Yang *et al.* prepared P-MXene/Fe_3_O_4_@MS magnetic sponge, as shown in Fig. 6d, that used solar-induced heating to markedly reduce crude-oil viscosity, enhancing absorption beyond 27 fold its weight and enabling controlled manipulation *via* magnets. While not exclusively MXene based, it establishes the quantitative benefit of photothermal heating for heavy-oil remediation that MXene sorbents harness similarly. The concept of solar-tuned viscosity reduction to speed up uptake and drainage translates directly to MXene-coated foams/aerogels. This work provides a benchmark for evaluating MXene photothermal efficacy.^131^

### 3.4 Magnetically responsive MXene systems

**Fig. 7:**
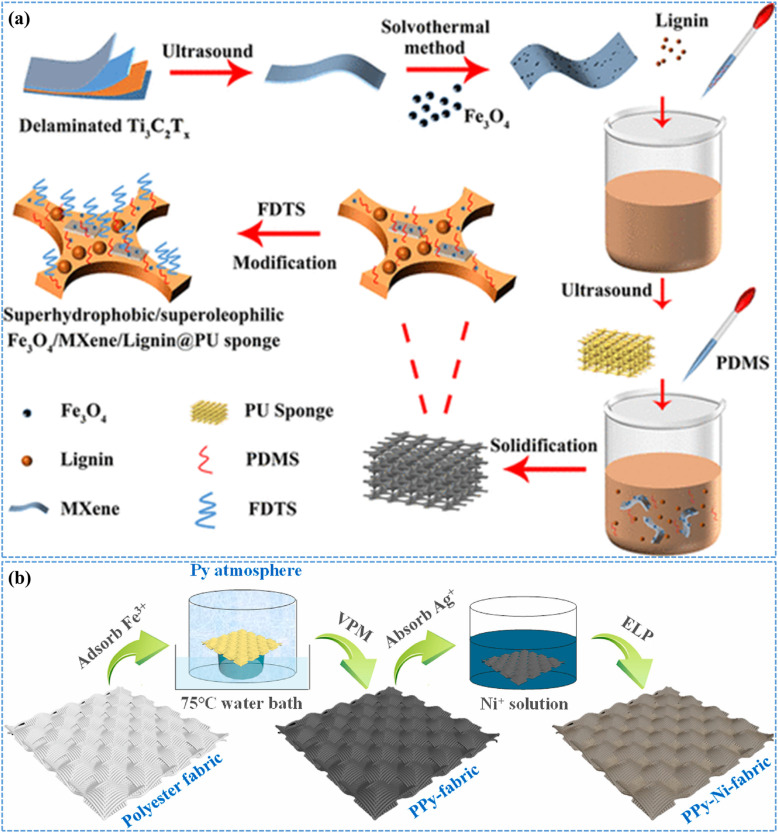
(a) Schematic of the synthesis route for the Fe_3_O_4_/MXene/lignin@PU sponge. Reproduced with permission from ref. 142. Copyright 2023, the American Chemical Society. (b) Construction of a conductive fabric membrane coated with polypyrrole–Ni. Reproduced with permission from ref. 148. Copyright 2021, Elsevier.

Embedding magnetic particles into MXene structures creates smart sorbents that can be remotely manipulated by external fields. From a mechanistic standpoint, magnetic responsiveness does not directly influence separation selectivity but instead enables external control over sorbent positioning and recovery.^132,133^ The separation process itself remains governed by wettability and capillary-driven absorption, while magnetic forces provide a means for rapid retrieval and redeployment. In some cases, the incorporation of magnetic nanoparticles also modifies surface roughness and interfacial energy, indirectly affecting wettability and enhancing oil affinity.^134,135^ Furthermore, when combined with photothermal MXene components, magnetic systems can exhibit synergistic effects, where localized heating reduces oil viscosity while magnetic actuation improves transport and collection efficiency. Usually, superparamagnetic iron oxides (Fe_3_O_4_ or γ-Fe_2_O_3_ nanoparticles) are synthesized *in situ* on Ti_3_C_2_T_*x*_ sheets, or deposited on MXene/polymer supports.^136^ The resulting Fe_3_O_4_–MXene composites exhibit oil affinity with facile recovery; once oil is adsorbed, material can be extracted from water using a simple magnet.^137,138^ This magnetic responsiveness greatly facilitates separation and reuse. Additionally, magnetic MXene systems may perform other functions; for instance, particles of Fe_3_O_4_ tend to enhance light absorption, providing photothermal heating under sunlight and subsequently improving the rate of oil recovery.^139^ The magnetic properties of the sponge enabled it to be moved and positioned using magnets for oil-water separation. This shows that MXene-Fe_3_O_4_ materials can be used to integrate magnetically enabled deployment with solar- and electro-thermal oil recovery. In both cases, MXene provided electrical and thermal conductivity and oil affinity, whereas Fe_3_O_4_ provided magnetic responsiveness and photothermal absorption.^140^ These magnetically responsive MXene systems offer clear advantages, including easy recovery and reuse, since a magnet can collect the spent sorbent without the need for filtration, as well as mobility and targeted deployment.^130,141^ Magnetic particles can also catalyze secondary reactions (Fenton-like oxidation of organics under light), although in practice this effect has been less explored in oil clean-up.^120^ By contrast, ordinary sponges or carbon sorbents must be physically removed, a slow process that is prone to material loss or fragmentation. Magnetized MXene composites avoid such losses and can be cleaned (*e.g.*, by solvent or thermal treatment) and redeployed. The recent literature highlights these advantages, for instance, Guo *et al.* reported a polyurethane (PU) sponge decorated with an Fe_3_O_4_/MXene/lignin composite (Fig. 7a). The native hydrophobic PU was coated with lignin (a biopolymer), Ti_3_C_2_T_*x*_ nanosheets, and Fe_3_O_4_ nanoparticles, yielding a sponge that was superhydrophobic (water contact angle of 145°) and superoleophilic. Critically, the Fe_3_O_4_/MXene–lignin coating was magnetic, and after adsorption, the sponge could be manipulated by magnets, enabling its easy retrieval or even self-floating arrangement. Under simulated sunlight, the composite achieved photothermal heating up to 64 °C on its surface, boosting its oil recovery rate by 27% (flux of 35 469 L m^−2^ h^−1^ for viscous oil) compared to dark conditions. This work exemplifies how magnetic MXene composites can integrate high separation efficiency (98% oil rejection) with simple handling and reusability (maintaining a capacity of 69.6 g g^−1^ after 15 cycles).^142^ Similarly, Yang *et al.* prepared a magnetic Ti_3_C_2_T_*x*_ sponge for crude oil spill clean-up. A melamine foam was sequentially coated with Ti_3_C_2_T_*x*_, Fe_3_O_4_ nanoparticles and polydimethylsiloxane (PDMS). The resulting P-MXene/Fe_3_O_4_@MS sponge was highly hydrophobic (water contact angle of 147°) and strongly magnetic. Under 1 sun illumination, the temperature of the sponge increased to 84 °C, and upon application of a 20 V bias for Joule heating, it reached 100 °C, drastically reducing the viscosity of crude oil. In tests, the magnetic sponge absorbed >27 times its weight in crude oil within 2 min under illumination, and a pump-assisted device using this sponge achieved continuous removal of viscous oil (flux of 710 kg m^−2^ h^−1^).^143^

### 3.5 Electro-assisted/capacitive emulsion separation

It should be noted that experimental demonstrations of MXene-based electro-assisted oil–water separation systems remain limited. Therefore, the following discussion distinguishes between experimentally validated observations and conceptual mechanisms inferred from analogous electrochemical systems. Electro-assisted techniques exploit electric fields and electrode interfaces to destabilize emulsions or adsorb charged species. In a typical configuration, electrodes are submerged in the emulsion and a DC or pulsed voltage is applied. The electric field induces dielectrophoretic forces on oil droplets (causing coalescence), drives electrolysis reactions at the electrodes (generating bubbles that carry oil out), or charges the droplet–electrode interface, thereby pinning droplets onto the electrode surfaces.^144,145^ Capacitive deionization (CDI) is a technique for ionic separation, in which porous electrodes (often carbon) are charged, creating electrical double layers that adsorb ions. For emulsions, a charged electrode might likewise attract polar oils or ionized surfactants at droplet surfaces, weakening the emulsion.^146^ MXenes are ideal electrode materials for these processes, as their high electrical conductivity and large specific capacitance (due to layered structure and surface terminations) facilitate high charge storage and fast charge–discharge cycles. A Ti_3_C_2_T_*x*_ electrode can store significant ionic charge at moderate voltages (*e.g.*, 1–2 V) and thus generate strong electric fields in its vicinity.^147^ Although relatively few studies have explicitly demonstrated MXene electrodes for oil emulsions, analogies can be drawn from related work. As a conceptual analogue, Li *et al.* constructed a conductive fabric membrane coated with polypyrrole–Ni (Fig. 7b) (not involving MXene).^148^ This electroflotation concept would be further enhanced if the cathode were an MXene-based mesh, where Ti_3_C_2_T_*x*_ could serve as a stable conductive substrate for metal catalysts while resisting fouling. Similarly, it has been noted that charging a membrane surface can change its wettability in principle; in this context, an MXene film under potential might switch from hydrophilic to hydrophobic, enabling electrically tunable oil sieving.^149^ In capacitive setups, Ti_3_C_2_T_*x*_ electrodes have been shown to achieve high specific capacitance (hundreds of F g^−1^) in desalination cells. Analogously, an electrode cell with MXene anodes and cathodes immersed in an oil–water emulsion could draw oil droplets toward the electrode surface *via* induced dipole interactions or by capturing charged surfactant layers. Although these ideas are still emerging, MXene performance in analogous electrochemical applications (supercapacitors and CDI) suggests strong potential. The advantages of MXenes for electro-assisted separation include ultrafast charge transfer and high electrochemical surface area.^150^ Under an applied electric field, Ti_3_C_2_T_*x*_ electrodes can quickly redistribute charge along their sheets, generating strong local fields that can polarize droplets and promote droplet–droplet attraction (electrocoalescence). The ability of MXenes to intercalate ions enables the formation of electric double layers, which may physically attract polar molecules on the droplet surface. MXene electrodes can also facilitate electrochemical oxidation of dissolved oils at high potentials, partially integrating separation with degradation. Because MXenes are inherently hydrophilic (–OH and –O groups), they readily wet water and provide good contact with the liquid phase, unlike some hydrophobic carbon electrodes. However, there are serious limitations. Pure water is an insulator, and a source of electrolyte (*e.g.* salts) is required to conduct current, which in practice necessitates the addition of chemicals to the wastewater. The electrode may become electrically insulated due to surface fouling by oil or organics, or its pores may become blocked, necessitating frequent cleaning.^151^ The durability of MXenes under an applied bias is also an issue, as MXenes may oxidize to titanium oxides at anodic potentials, thereby altering their properties. Another problem is energy efficiency: in contrast to passive membranes, electro-assisted separation requires external power, and the overall economics depend on the applied voltage and processing time. When oil droplets have relatively small net charge, their response to uniform fields can be weak, and thus novel electrode designs (*e.g.*, patterned fields) are required. The strategies for improvement should focus on electrode architecture (*e.g.*, porous 3D MXene foams to increase active area), bipolar electrode configurations to concentrate fields, and pulsed or alternating current operation to avoid charge accumulation. Coating MXenes with electrocatalysts (*e.g.*, Pt and Ni) could lower overpotentials for gas evolution, generating microbubbles that entrain oil (electroflotation). Coupling fields with membranes (electrofiltration), *i.e.* applying a field across an MXene-based nanofiltration membrane, could enhance rejection through electrophoresis. Also, pairing MXene electrodes with UV illumination (photoelectrochemistry) might allow simultaneous electrocoagulation and photocatalysis. On the materials front, preparing MXene derivatives with enhanced electrochemical stability (*e.g.* doped Ti_3_C_2_, or Nb/C-based MXenes) could reduce degradation.

Electro-assisted oil–water separation using MXene materials remains an emerging area, with much of the current understanding derived from related electrochemical systems such as capacitive deionization and electroflotation. Although MXenes offer clear advantages in conductivity and surface functionality, further experimental validation is required to establish definitive mechanistic pathways and assess their practical applicability in emulsion separation systems.

In summary, MXene-based oil–water separation is governed by multiple interacting mechanisms, including wettability-controlled interfacial rejection, nanochannel-mediated transport, capillary-driven absorption, and externally stimulated processes such as photothermal or electro-assisted effects. The dominant mechanism depends strongly on emulsion characteristics, particularly droplet size distribution, surfactant chemistry, and operating conditions. A clear distinction between these mechanisms is essential for rational design and accurate interpretation of separation performance (Table 2).

**Table 2:** Summary of reported studies on MXene-based membranes and their performance metrices for oil-water separation

Composite	Type	Oil type	Flux (L m^−2^ h^−1^)	Efficiency (%)	Number of cycles	References
TA-APTES@MXene	Membrane	Petroleum ether, lubricating oil and vegetable oil	5990.11	98	4	152
Na-bentonite@MXene	Membrane	Cyclohexane, lubricating oil and petroleum ether	1414	97	8	83
MXene@CS/TA-FeOOH	Membrane	Isooctane, *n*-hexane, petroleum ether, toluene, 1,3,5-trimethylbenzene, dichloromethane, and crude oil	1022.7	99.7	10	87
Hal@MXene-PDA	Membrane	Petroleum ether and lubricating oil	5036.2	99.8	3	153
PES-MXene@TiO_2_	Membrane	*n*-Hexane, cyclohexane, *n*-heptane, isooctane, petroleum ether, trichloromethane and diiodomethane	3045	99.8	10	154
TA-ZIF-8@MXene/CA	Membrane	Petroleum ether, lubricating oil and vegetable oil	5347.36	98	8	155
PDMS-Fe-MXene/A-HA	Aerogel	Pump oil	23 478	—	5	156
MXene/TiONT	Membrane	*n*-Hexane, petroleum ether, gasoline, and edible oil	578.7	99.7	10	157
MXene/PVDF nanofiber	Solar-driven membrane	Heavy mineral oil/saltwater (NaCl, 3.5%)	16 977.9	99	—	95
S–Fe_3_O_4_/MXene/lignin@PU	Sponge	*n*-Hexane, CCl_4_	35 469	98	15	158
PES-MXene MMMs	Membrane	Diesel	2280	98	—	159
MXene@COF-TpBD	Membrane	*n*-Hexane, *n*-heptane, isooctane, gasoline, and edible oil	1908.4	99.7	20	160
RGO/PDA/MXene	Composite-modified membrane	Petroleum ether	72.31411	97	6	161
MXene-BIC@MIL-101(Fe)	Photocatalytic self-cleaning membrane	*n*-Hexane, *n*-heptane, cyclohexane, petroleum ether, and isooctane	2534	99.7	10	162
Ti_3_C_2_T_*X*_ MXene-PAN	Membrane	*n*-Hexane, petrol, toluene, diesel and vegetable oil	1573	98.6	20	163
MXene-MWCNT	Photothermal membrane	Canola oil	80	95	4	2
MXene/epoxy sponge	Photothermal superwetting sponge	Thermal conductive oil, vacuum pump oil, soybean oil, engine oil, and *n*-hexane	3092.15	97.8	10	164
MS@PDMS/MXene/CSs	Photothermal superhydrophobic sponge	*n*-Hexane and CCl_4_	127 000	99.6	15	165
Mussel-inspired MXene-functionalized sponge	Photothermal adsorption	Crude oil	—	90	—	166
P-SBC/MXene	Photothermal aerogel	Crude oil	630	96.6	—	167
coPA/ML-Ti_3_C_2_T_*x*_	Membrane	Vegetable oil	10 000	99.8	—	168
MXene/3D-S-COF	Membrane	*n*-Hexane, petroleum ether, carbon tetrachloride, kerosene and diesel fuel	240	98	7	169
P-MXene/Fe_3_O_4_@MS	Heating-assisted absorption	Crude oil	710	—	10	131
Bi_2_O_2_CO_3_@MXene	Composite membrane	Lubricating oil and vegetable oil	815.3	99	5	170
MCE-MNRs	Membrane	Diesel, gasoline, edible oil and *n*-hexane	15 860	99	5	171
MTiO_2_-MXene	Composite membrane	*n*-Hexane	6815.85	99.8	64	172
ZnO/M-TA@PEN	Membrane	Petroleum ether, *n*-hexane, 1,3,5-trimethyl benzene, *n*-heptane, isooctane	2053.35	99.4	10	173
PVDF/Ti_3_C_2_/WO_3_·H_2_O	Membrane	Crude oil, petrol, *n*-hexane, and toluene	1542	99	10	174
WO_3_/MXene	Composite membrane	Bean oil, petrol, gear oil, xylene, or aniline	6.4	99.8	6	175
MXene@TiO_2_/PEN	Fibrous composite membrane	*n*-Hexane, petroleum ether, 1,3,5-trimethyl benzene, *n*-heptane and isooctane	1003	99	10	176

## 4. Sustainability and environmental risk assessment

**Fig. 8:**
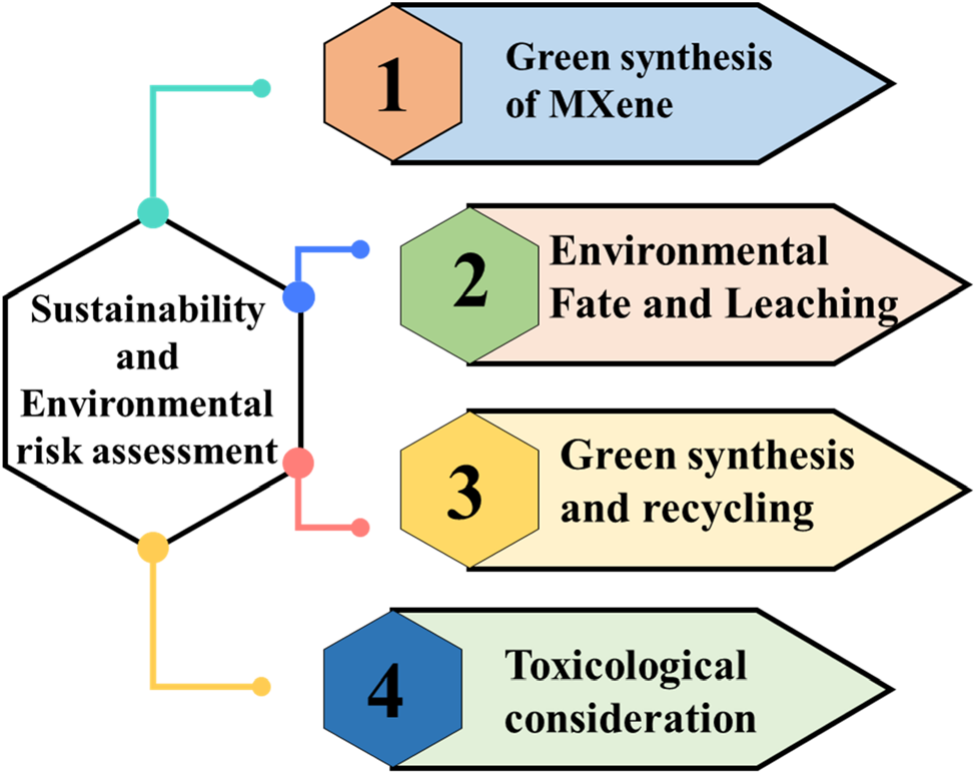
Sustainability and environmental risk assessment framework for MXene-based systems.

The application of MXene materials in oil–water separation raises important questions of sustainability, toxicity and life-cycle impact. MXenes are typically synthesized *via* hazardous etching (strong acids and fluoride salts) and often involve multi-step processes, and thus their green credentials demand scrutiny (Fig. 8).

### 4.1 MXene synthesis and environmental footprint

The conventional synthesis of Ti_3_C_2_T_*x*_ begins with the Ti_3_AlC_2_ MAX phase and involves strong etchants (*e.g.*, concentrated HF or HCl/LiF). These acids and their byproducts (AlF_3_ salts and spent etchants containing Al and Ti waste) must be neutralized and disposed of, generating chemical waste. Energy-intensive steps (high-temperature MAX sintering and ultrasonication delamination) further increase the environmental footprint. Quantitative life-cycle assessments (LCAs) highlight the cost. Dadashi Firouzjaei *et al.* performed a cradle-to-gate LCA of lab-scale Ti_3_C_2_T_*x*_ synthesis for EMI shielding applications. They found that producing 1 kg of MXene (*via* conventional HF etching) emits ≈428 kg CO_2_-equivalent, an order of magnitude higher than producing 1 kg of aluminum foil (23 kg CO_2_) or copper foil (8.75 kg CO_2_).^177^ Electricity consumption (for furnace heating and sonication) contributed more than 70% of the effect, while chemicals (HF, LiF, and HCl) contributing less. Simultaneously, Hansen *et al.* used a Safe-and-Sustainable-by-Design framework to the synthesis of Ti_3_C_2_T_*x*_ and concluded that titanium powder synthesis (used for the MAX phase precursor) is the only hotspot in the life cycle of MXenes.^178^ These discussions highlight the fact that, as it is practiced, MXene production is carbon-intensive and requires critical minerals (Ti, Al, Li, and F). By comparison, traditional oil cleanup materials such as polypropylene mats or activated carbon have their own burdens (fossil feedstocks and high-temperature carbonization) but generally lower energy footprints per kilogram.

### 4.2 Green synthesis and recycling

A quantitative comparison between conventional HF-based etching and emerging fluorine-free synthesis routes reveals critical differences across three environmental performance dimensions. In terms of energy consumption, Dadashi Firouzjaei *et al.*^177^ reported that conventional HF/LiF etching of Ti_3_C_2_T_*x*_ consumes sufficient energy to generate approximately 428 kg CO_2_-equivalent per kilogram of MXene produced, with electricity accounting for over 70% of this burden due to high-temperature MAX phase sintering and prolonged ultrasonication. By contrast, electrochemical delamination and molten-salt fluorine-free routes operate at significantly lower temperatures and shorter processing times, reducing energy-related emissions by an estimated 40–60% based on comparative process analysis.^179^ Regarding COD emissions, conventional HF etching generates fluorine-containing acidic effluents with high chemical oxygen demand, requiring neutralization with large volumes of alkaline solution and producing AlF_3_ sludge as a byproduct, which constitutes a secondary contamination stream. Fluorine-free alkali-based routes such as hydrothermal KOH etching eliminate fluorine byproducts entirely, substantially reducing effluent COD and eliminating the need for fluoride waste management.^178^ However, alkali-based routes introduce high-pH waste streams that themselves require neutralization, representing a trade-off rather than a complete environmental solution. With respect to MXene recovery and yield, HF-based methods typically achieve higher delamination yields and more uniform flake size distributions, whereas fluorine-free methods currently suffer from lower yields and less controlled morphologies, which indirectly increase the environmental burden per unit of functional MXene produced.^177^ Ungureanu *et al.*^179^ further demonstrated through systematic LCA comparison that titanium powder synthesis remains the dominant environmental hotspot regardless of etching route, suggesting that upstream MAX phase production must also be addressed alongside etchant substitution to achieve meaningful life-cycle improvement. These quantitative distinctions establish that fluorine-free synthesis offers clear advantages in effluent toxicity and worker safety but does not yet represent a complete solution to the environmental footprint of MXene production, and that recovery efficiency must improve before fluorine-free routes become unambiguously superior on a full LCA basis.

### 4.3 Toxicological consideration

The intrinsic toxicity of MXenes has been the subject of several preliminary studies. In vitro assays (on mammalian cell lines) have indicated that delaminated Ti_3_C_2_T_*x*_ exhibits low to moderate cytotoxicity and generally does not cause acute cell death at concentrations of tens of µg mL^−1^, although reactive oxygen species generation has been observed at high doses. More relevantly, aquatic ecotoxicity has been evaluated with model organisms. Nasrallah *et al.* exposed zebrafish embryos to Ti_3_C_2_T_*x*_ suspensions and found an LC_50_ of 257 µg mL^−1^ (at 96 h). No developmental abnormalities were seen below 100 µg mL^−1^, and locomotor functions were unaffected at 50 µg mL^−1^.^180^ According to the US Fish and Wildlife Service acute toxicity criteria, concentrations <100 µg mL^−1^ are classified as practically non-toxic. Similarly, Hansen *et al.* observed that *Daphnia magna* and freshwater algae exhibited no significant acute toxicity up to 100 mg L^−1^ of Ti_3_C_2_T_*x*_ (EC_50_ > 100 mg L^−1^). The presence of humic acids (environmental natural organic matter) did not appreciably alter MXene toxicity.^178^ These findings suggest that Ti_3_C_2_T_*x*_, at likely exposure levels in nature, poses low immediate hazard to aquatic life. The potential toxicity of oxidation by-products (*e.g.*, if Ti_3_C_2_T_*x*_ gradually converts to nanoparticulate TiO_2_) is also a concern, although bulk TiO_2_ exhibits low toxicity in aquatic systems. Very few studies exist on the immunological or long-term subcellular effects of MXenes. More specific experimental evidence on the toxicity of MXene degradation products, particularly TiO_2_ nanoparticles formed through the progressive oxidation of Ti_3_C_2_T_*x*_ in aquatic environments, warrants careful consideration. TiO_2_ nanoparticles generated from MXene oxidation are predominantly in the anatase phase and typically range from 5–20 nm in diameter, a size range known to exhibit greater biological reactivity than bulk TiO_2_ due to their higher surface area and enhanced reactive oxygen species (ROS) generation under UV irradiation.^181^ The reported LC_50_ values for anatase TiO_2_ nanoparticles in zebrafish embryo models range from 50–100 mg L^−1^ under dark conditions but decrease significantly to 1–10 mg L^−1^ under UV exposure due to photocatalytic ROS generation, indicating that environmental light conditions critically modulate degradation product toxicity.^182^ In *Daphnia magna*, chronic exposure to TiO_2_ nanoparticles at concentrations as low as 1 mg L^−1^ has been reported to reduce reproductive output by up to 35% over 21 day exposure periods, representing a sublethal effect not captured by standard acute toxicity endpoints.^183^ Regarding bioaccumulation, TiO_2_ nanoparticles have been shown to accumulate in the gills and intestinal epithelia of aquatic organisms, with bioconcentration factors reported between 10^3^ and 10^4^ in filter-feeding invertebrates, although active excretion mechanisms may limit long-term biomagnification through trophic levels.^184^ Furthermore, fluorine-containing surface termination byproducts released during MXene degradation, including fluoride ions and organofluorine species, may persist in aquatic environments and potentially exhibit endocrine-disrupting effects at concentrations exceeding 1.5 mg L^−1^, the WHO guideline value for fluoride in drinking water. Collectively, these data indicate that while the parent Ti_3_C_2_T_*x*_ phase exhibits low acute toxicity, its degradation products under environmentally realistic conditions present a more complex and potentially significant ecotoxicological risk profile that requires systematic long-term assessment rather than qualitative reassurance. In summary, the reported ecotoxicity for Ti_3_C_2_T_*x*_ is low (no acute hazard up to mg L^−1^), but comprehensive chronic and ecosystem-level assessments are still needed.

### 4.4 Environmental fate and leaching

The fate of MXene materials after use is another open question. As 2D titanium carbide, Ti_3_C_2_T_*x*_ is stable under neutral and basic conditions but slowly oxidizes in aqueous media to form TiO_2_ nanosheets, especially when exposed to air or strong oxidants. This transformation would likely immobilize the Ti into an oxide form but could release surface-bound species. For MXene composites (*e.g.*, MXene sponges), degradation over months could lead to fragmentation and slow release of Ti-containing particles. Experimental evidence suggests that Ti dissolution is negligible, but physical shedding of MXene flakes might occur with abrasion or extreme pH. In a chronic test, weeks of static water did not show any soluble Ti, suggesting that acute leaching of metal ions is insignificant.^179^ However, the ultimate mineralization to TiO_2_ raises the issue of bioaccumulation (TiO_2_ may be accumulated by organisms but is usually secreted). Carbon- and fluorine-based terminations on the surface can also be released into water, but Ti_3_C_2_T_*x*_ surfaces tend to lose –F terminations during washing, generating –OH/–O groups. Thus, the fate of any exfoliation or delamination agent (Li^+^ and urea) should be considered and regulated.

### 4.5 Green synthesis and recommendations

To shift to greener MXene production and use, a series of strategies are recommended. Replacement of HF with milder etchants (*e.g.* alkali fusion, ammonium bifluoride or electrochemical delamination) would remove fluorine by-products and minimize the risk to workers. Emissions can be restricted by adopting closed-loop systems that recycle etchants and neutralize fumes. Heating/sonication with renewable energy would reduce the overall carbon footprint, which is based on electricity use. To implement this, MXene oil traps that can be cleaned and reused multiple times should be designed, which will offset the initial production cost. MXenes possess strong capabilities to separate oily wastewater, but their environmental suitability requires careful design and development of materials. It is therefore vital to continue the discovery of green MXene synthesis routes alongside systematic risk assessment frameworks (life cycle assessment and SSbD approaches). Until then, practitioners should treat MXenes with caution, using them where their high performance (*e.g.* self-cleaning, magnetism) outweighs production impacts, and ensuring robust handling (containing spills of nanomaterials, recycling solvents, *etc.*). By combining innovation in MXene chemistry with principles of green engineering, it is plausible to harness their benefits for oil–water separation while keeping environmental and health risks low.

### 4.6 Oxidative stability and long-term operational considerations

The susceptibility of Ti_3_C_2_T_*x*_ to oxidation in aquatic environments represents the most critical barrier to its industrial deployment; therefore, a rigorous comparative evaluation of available stabilization strategies is essential. Four principal modification approaches have been reported, each with distinct advantages and limitations. Antioxidant additives, such as sodium ascorbate or imidazolium-based compounds dispersed within MXene colloidal suspensions, offer a simple and low-cost stabilization route that can demonstrably retard surface oxidation kinetics. However, this approach provides only temporary protection as antioxidants are consumed over time and are not covalently bound to the MXene surface, making them unsuitable for long-term operational stability in continuous flow systems where antioxidant depletion cannot be practically monitored or replenished.^185,186^

Surface coating strategies, including polymer encapsulation with PVDF, PVA, or polyimide layers, provide more durable oxidative protection by physically blocking oxygen and water access to carbide edges and surface terminations. These coatings have maintained MXene conductivity and structural integrity over extended periods under ambient aqueous conditions. However, polymer coatings inevitably reduce the accessible surface area and may partially block nanochannel transport pathways, introducing a direct trade-off between oxidative protection and separation flux performance.^187,188^ Hybrid nanocomposite design, where MXene is embedded within carbon-based matrices such as graphene or CNT networks, offers dual benefits of oxidative shielding through physical encapsulation and structural reinforcement that resists delamination under cyclic hydraulic stress. However, this approach introduces fabrication complexity and cost, and the long-term stability of the MXene-carbon interface under acidic or alkaline industrial effluents remains insufficiently characterized.^54^ Intercalation and crosslinking strategies, such as sodium alginate or clay intercalation between MXene layers, stabilize the interlayer spacing and restrict water ingress to oxidation-prone edge sites. These approaches have demonstrated pH-stable operation across pH 3–10 in short-term studies. However, critically, the majority of reported stability assessments span only days to weeks under laboratory conditions, with very few studies evaluating performance degradation over operationally relevant timescales of months to years under real industrial effluent chemistry including high ionic strength, mixed surfactants, and variable temperature conditions.^38,83^ Across all four strategies, a fundamental gap exists: long-term operational stability data under industrially realistic conditions are largely absent from the literature. This represents a critical unresolved challenge that must be systematically addressed through standardized accelerated aging protocols and extended pilot-scale testing before MXene-based separation systems can be considered viable for industrial deployment.^177,179^

## 5. Challenges, opportunities, and future directions

### 5.1 Challenges

**Fig. 9:**
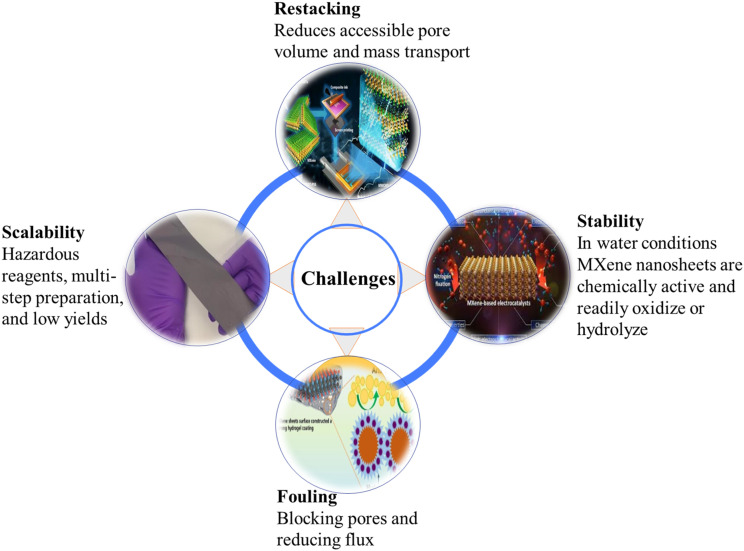
Several challenges associated with MXenes.

MXene-based membranes (*e.g.* Ti_3_C_2_T_*x*_) exhibit exceptional oil–water separation performance, but several inherent challenges limit their practical use. Here, we discuss five key issues, including oxidative stability, nanosheet restacking, scalability/cost, fouling and degradation, and disposal and regeneration, with relevant literature citations (Fig. 9). For each challenge, we also outline the reported mitigation strategies or future directions.

#### 5.1.1 Stability of MXenes in oxidative environments

MXene nanosheets (especially Ti_3_C_2_T_*x*_) are chemically active and readily oxidize or hydrolyze, which can deteriorate membrane performance. Under aqueous or oxygen-rich conditions, their surface terminations (–O, –OH, and –F) and exposed carbide edges react to form TiO_2_ and other oxides. This intrinsic oxidation severely limits the long-term structural integrity and separation efficiency of MXene membranes.^2,38^ Indeed, recent reviews note that oxidation and related hydrolysis constitute a major practical challenge for MXene membranes, compromising their durability.^2^ For example, Younas *et al.* observed that narrow interlayer spacing and facile oxidation severely limit mass diffusion and lead to poor stability in MXene separation layers.^38^ Thus, while MXenes are intrinsically hydrophilic and chemically reactive (attributes desirable for oil–water separation), uncontrolled oxidation of Ti_3_C_2_T_*x*_ threatens membrane lifespan and performance.

Mitigation strategies: Various strategies have been suggested to shield MXenes from oxidation. Among these, surface passivation strategies, such as depositing a thin layer of polymer, oxide or any other film onto MXene sheets, serve as protection against O_2_ and H_2_O.^187,188^ The dispersion of antioxidant additives (*e.g.*, sodium ascorbate or imidazolium salts) has been demonstrated to inhibit the oxidation of Ti_3_C_2_T_*x*_ colloids.^185,186^ Another method is the design of hybrid nanocomposites, for example, using MXene as a matrix containing antioxidants or carbon-based materials.^54^

#### 5.1.2 Restacking of nanosheets in membranes

In MXene membranes, the individual 2D sheets are prone to restacking or aggregation during fabrication, which reduces the available pore volume and mass transport. For example, vacuum-filtration assembly typically results in dense Ti_3_C_2_T_*x*_ laminates. This leads to irregular stacking of layers, forming non-uniform nanochannels and unwanted inter-layer pores. Self-restacking of these layers reduces the effective interlayer spacing, and permeability is reduced dramatically. Huang *et al.* observed that the stacking of nanosheets in MXene laminates results in nonuniform sub-nanometer channels and defects, which directly affect their separation performance.^56^ In practice, restacking may severely impair membrane performance during operation, as water transport channels are squeezed or blocked by overlapping sheets. Therefore, excessive restacking should be avoided to maintain the high flux and selectivity of MXene membranes.

Mitigation strategies: To prevent restacking, scientists introduce interlayer spacers or fabricate mixed-matrix composites.^189^ One of these is to insert large, rigid molecules or nanoparticles between MXene sheets. Similarly, the incorporation of MOF or ZIF particles has been shown to prevent restacking of MXene (the porous MOF particles serve as the spacer).^190^ Another approach is mixed-matrix membranes (MMMs): MXene sheets are dispersed within a polymer matrix to prevent them from lying flat and densely packing.^191^

#### 5.1.3 Scalability and cost of synthesis

One of the major drawbacks of MXene membranes is that producing large-area, defect-free films is complex and costly. Traditionally, MXenes (*e.g.* Ti_3_C_2_T_*x*_) are prepared *via* HF etching of the MAX phase using a top-down approach. This process requires toxic fluorine substances and prolonged ultrasonication, making it lengthy and difficult to scale. In addition, the standard etchants typically delaminate the material only partially, thus requiring multiple steps or intercalants, which increases the cost and variability further. Similarly, vacuum-filtration assembly of MXene membranes is a lab-scale batch reaction, which does not readily replicate to roll-to-roll or industrial manufacturing. In short, it is challenging to produce uniform and reproducible MXene membranes on a large scale. In recent reports, the authors clearly mention that the controllability, reproducibility and scalability of MXene membrane synthesis should be improved. The hazardous reagents, multi-step preparation and low yields associated with these hurdles result in high cost and minimal throughput.

Mitigation strategies: New research directions focus on the simplification of MXene preparation and membrane fabrication. One approach is the use of milder, fluorine-free etchants. For example, Ghidiu *et al.* designed an *in situ* etching technique with LiF/HCl as an alternative to concentrated HF. Despite its weaker chemistry, high-quality Ti_3_C_2_T_*x*_ (with the advantage of increased interlayer spacing and faster exfoliation) can be obtained with reduced hazard.^192^ Other studies explore NH_4_HF_2_ or molten-salt etching, and even bottom-up synthesis (CVD or templating) for scalable 2D carbides.^193–195^ On the membrane side, mixed-matrix approaches can reduce costs; for example, embedding a small fraction of MXene in a cheap polymer can achieve high performance while using much less MXene.^196,197^ These MMMs use standard coating or casting techniques (*e.g.*, blade coating or roll-to-roll processing) for scalability while leveraging the unique chemistry of MXenes.

#### 5.1.4 Fouling and degradation over time

Similar to all separation membranes, MXene membranes suffer from fouling by oil and other contaminants, which leads to performance degradation upon reuse. In oil–water separation, hydrophobic oil droplets tend to adhere to the membrane surface, blocking pores and reducing flux. Even highly hydrophilic MXene surfaces can be fouled, as viscous oil can fill interlayer channels or coat the sheet edges, leading to a rapid decline in flux. For example, the oil accumulation within pores (pore plugging) can reduce the permeate flux to well below 10% of the original value.^198–200^ Prior studies on oil–membrane interactions also emphasize that heavy oils strongly adhere the membrane surfaces and cause irreversible fouling, preventing membrane reuse. In practice, fouling manifests as declining flux and eventual membrane failure after repeated cycles, especially with dirty or viscous feed. Maintaining flux and selectivity in numerous cycles is one of the challenges facing MXene membranes in oily wastewater treatment.

Mitigation strategies: Various strategies have been investigated to overcome fouling. One of the ways that has been tested is to ensure that the surface is very antifouling. For example, incorporating superhydrophilic/underwater-oleophobic materials (*e.g.*, TiO_2_ nanoparticles, zwitterionic polymers or hydrophilic PVA) forms a slip layer that prevents oil adhesion.^201,202^ Another tactic is photothermal self-cleaning, in which MXenes strongly absorb infrared/visible light and convert it into heat.^203^ By designing a composite MXene membrane that heats up under sunlight or an IR lamp, adhered oil can be locally mobilized or removed *via* evaporation. Finally, mechanical and chemical cleaning protocols (*e.g.*, backwashing, solvent rinsing, or mild surfactant cleaning) can be used to periodically regenerate membranes.

### 5.2 Directions in MXene-based oil–water separation

**Fig. 10:**
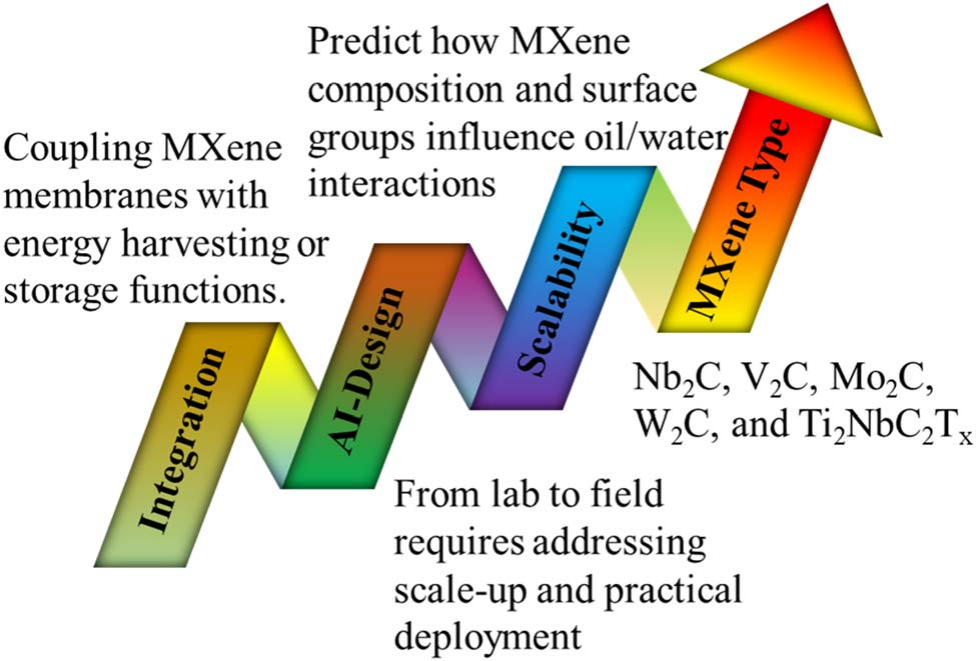
Directions provided for future development.

This section focuses on the emerging strategies aimed at improving the long-term stability and scalability of MXene-based systems for oil–water separation (Fig. 10). Particular emphasis is placed on material engineering approaches and system-level optimizations that address current limitations under realistic operational conditions.

#### 5.2.1 Energy-integrated oil–water separation

Recent efforts have explored coupling MXene membranes with energy harvesting or storage functions. The strong photothermal response of MXenes enables solar-driven or thermal energy-assisted separation and self-cleaning. For example, photothermal MXene–PVDF nanofiber membranes can generate steam under 1 sun illumination while rejecting oil, and a prototype device achieved a water evaporation rate of 1.6 kg m^−2^ h^−1^ and an extremely high oil flux (1.7 × 10^4^ L m^−2^ h^−1^ for hexane) under low vacuum conditions.^204^ In related work, MXene-functionalized sponges and aerogels have been shown to harness solar energy; for example, a mussel-inspired Ti_3_C_2_T_*x*_-coated sponge showed efficient crude-oil uptake and solar steam generation for water purification (photocatalytic sterilization and evaporation) under sunlight.^166^ MXene membranes have also been combined with photocatalysts, where incorporating N-doped Bi_2_O_2_CO_3_ or β-FeOOH into MXene laminates creates 2D/2D heterojunctions that both separate oil–water emulsions (flux reaching 500.38–1022.7 L m^−2^ h^−1^ at >99% rejection) and degrade organic pollutants under visible light. After fouling, light-driven photocatalysis can fully restore membrane flux, enabling long-term operation.^87^ Beyond photothermal and photocatalytic effects, MXene systems are being designed to capture other waste energies during separation. For instance, the electrical conductivity and ion-sieving properties of MXenes can enable blue energy generation, and recent reviews have highlighted their suitability for hydrovoltaic and osmotic power harvesting (*via* water flow or salinity gradients) alongside separation.^205^ Thus, advanced MXene devices aim for all-in-one functionality; for example, a membrane that absorbs oil and uses thermal energy (solar or waste heat) for regeneration or energy harvesting, or even acting as an electrode in a supercapacitor or battery to store energy from flowing fluids. Conceptual designs have been proposed for MXene membranes integrated into energy-storage cells, leveraging the pseudocapacitance of MXenes. While full devices are still at an early stage, high-impact studies have begun to demonstrate these principles.

#### 5.2.2 Computational and AI-guided design

The design of MXene membranes increasingly relies on modeling and machine learning. Atomistic simulations (DFT and MD) have been used to predict how MXene composition and surface groups influence oil/water interactions. First-principles studies show that terminations such as –OH and –O create strong hydrogen bonding with water, whereas –F or –Cl reduce hydrophilicity.^206^ Molecular dynamic simulations of flowthrough model MXene nanochannels reveal ultrafast water permeation and high salt rejection (even under reverse-osmosis pressures) for sub-nanometer pores. MD simulations also help quantify fouling; for example, simulations of oil–water mixtures indicate that interfacial structuring of oil on MXene surfaces depends on substrate charge and roughness. Although direct oil-water MD studies on MXenes are scarce, analogies from MD simulations of graphene-based membranes and MXene desalination can guide membrane design. In practice, simulations use software such as VASP or Quantum ESPRESSO for DFT calculations (*e.g.*, computing MXene surface energies and adsorption energies), and LAMMPS or GROMACS with reactive force fields for MD transport simulations. These tools help screen candidate MXenes (layer composition, spacing, and terminations) before experimental tests.

Critically, machine learning (ML) and AI are accelerating MXene materials discovery. High-throughput DFT databases enable ML models to predict MXene properties, for example, Xiao *et al.* screened >600 MXenes using graph neural networks and DFT to identify novel out-of-plane piezoelectrics. Other ML efforts have trained DFT datasets to predict MXene electrical/ion–transport properties, guiding selection for supercapacitors or sensors. Beyond screening, active learning systems have also been applied; for example, a study reported a closed-loop robotic synthesis of MXene–nanocellulose aerogels, in which ML algorithms jointly optimized elastic modulus and electrical resistance.^207^ In this case, Gaussian processes and multi-objective Bayesian optimization steered thousands of experimental iterations, enabling the discovery of aerogel formulations that simultaneously improve both strength and conductivity.

Several specialized software tools support MXene modeling. Materials informatics frameworks (*e.g.* pymatgen and MAPLE) enable the calculation of formation energies and exfoliation energies across MXene compositions. For membrane performance, continuum modeling and computational fluid dynamics (CFD) are emerging approaches; for example, coupling MD-derived slip lengths with macroscale flow models to predict flux in module-scale membranes. In parallel, AI-driven materials design platforms (*e.g.*, Materials Project, NOMAD, or custom MXene repositories) and generative models (*e.g.*, variational autoencoders) are being explored to suggest new MXene chemistries or composites.

These computational advances allow the *in silico* design of MXene filters: DFT/MD identify promising terminations and pore structures, while ML accelerates materials screening and process optimization. For example, simulations indicate that nanoporous Ti_2_C or Nb_2_CO_2_ with customized surface terminations may be more effective than Ti_3_C_2_T_*x*_ in terms of permeation or selectivity (although this has not yet been experimentally proven). Next-generation AI models may be able to predict fouling behaviour and membrane lifetime by learning from simulated and experimental data, which will aid in the design of MXene membranes with optimal oil repellency and flux.

#### 5.2.3 Emerging MXene families for oil–water separation

Beyond the prototypical Ti_3_C_2_T_*x*_, a new generation of MXenes is being explored to enhance separation performance. Transition-metal carbides and nitrides such as Nb_2_C, V_2_C, Mo_2_C, and W_2_C, and double-transition MXenes (*e.g.*, Ti_2_NbC_2_T_*x*_) offer different chemistries and surface charge characteristics.^31^ These MXenes may exhibit different hydrophilicity, stability or catalytic activity. For instance, V_2_CT_*x*_ (vanadium carbide) has been fabricated into porous membranes.^208^ Although quantitative comparisons are scarce, qualitative trends are emerging, and early experiments suggest that some non-Ti MXenes can match or exceed Ti_3_C_2_T_*x*_ in terms of underwater oleophobicity when properly terminated. The other important direction is surface termination engineering. Historically, MXenes made by HF etching are rich in –OH and –O terminations (super-hydrophilic), although new synthesis methods have produced exotic variants: molten-salt etching forms -Cl-terminated MXenes, while post-selenisation or sulfurisation can be used. These groups dramatically alter surface wettability. For instance, Cl-terminated MXene films have higher water contact angles and enhanced oil repellency than F- or O-terminated analogs (due to the larger, less polar Cl, which reduces surface energy). Likewise, –S or –Se terminations (or doping with MoS_2_-like layers) create surfaces that can switch between superhydrophilic and superoleophilic states upon exposure to light or voltage. Thus, tuning terminations offers a way to tailor membrane selectivity; for example, a Cl-rich surface may be useful for hydrophobic coating layers that let oil pass but hold water, while an –OH-rich surface forms a hydration layer that repels oil (Table 3).

**Table 3:** Selected MXene materials and terminations for oil–water separation

MXene composition	Typical terminations	Wettability/notes	Separation performance
Ti_3_C_2_T_*x*_ (Ti_3_C_2_O_2_, OH)	–O, –OH (hydrophilic)	Superhydrophilic; UOCA up to 151° (with alginate).^87^	Lab membranes show >99% oil rejection, flux 10^3^ L m^−2^ h^−1^ (ref. 171)
Nb_2_CT_*x*_	–O, –OH (presumed)	Predicted intrinsically hydrophilic	Under study, expected high chemical stability
V_2_CT_*x*_	–O, –OH (presumed)	Hydrophilic; known for high mechanical toughness	Shows robust separation
Mo_2_CT_*x*_	–O, –OH or –S	Tunable *via* S–S bonds; potential photocatalysis	No data reported for oil yet; MoOx may add hydrophilicity
Ti_2_NbC_2_T_*x*_ (double M)	Mixed –O/–OH/F	Combines properties of Ti and Nb; possibly intermediate wettability	Could combine benefits of Ti_3_C_2_ and Nb_2_C (DFT predicts high conductivity)

**Surface terminations**			
–Cl (Cl-terminated)	–Cl	Lower surface energy than –OH; more hydrophobic (higher water CA)	Molten salt–synthesized MXene showed enhanced oil resistance (normative)
–F (F-terminated)	–F	Hydrophilic but less so than –OH; often present after HF etch	Common in HF-derived MXene; oil-adhesion is still very low due to hydration
–S, –Se (chalcogen)	–S, –Se	Potentially hydrophobic or switchable	Varying reports: –S terminations improved fluorocarbon lubricant adsorption
–OH/–O (native)	–OH, –O	Highly hydrophilic (water spreads)	Traditional MXene membranes are superwetting, enabling >99% rejection^2^.

## 6. Conclusion

MXene-based oil–water separation systems offer significant advantages over traditional materials. Their unique 2D chemistry, hydrophilicity, and charged surfaces with tunable nanochannels enable the fabrication of ultrathin membranes and highly porous sorbents that achieve 90–99% oil removal with exceptional water throughput. Hybrid composites further extend functionality, though a critical comparison across composite systems reveals that performance gains are highly context-dependent: carbon hybrids improve porosity and flux but may reduce mechanical robustness under cyclic compression; metal-oxide co-components boost photocatalytic self-cleaning but introduce fabrication complexity; and photothermal elements enhance viscous oil recovery but show variable efficiency depending on oil type and irradiation conditions. These trade-offs must be explicitly considered in material selection for specific application scenarios. Together, these features yield membranes and sponges with strong anti-fouling and self-cleaning characteristics, high reusability, and even smart responsiveness (magnetic or electrical actuation) for efficient spill cleanup and wastewater treatment. Nonetheless, practical implementation faces hurdles. MXene films are prone to oxidative degradation in water and restacking, which undermine stability and flux. Fouling by heavy oils and organics remains a challenge, as contaminants can block nanochannels and reduce permeation over time. The scalability of MXene production and membrane fabrication is also limited by complex synthesis (including the use of toxic etchants and high energy consumption) and batch processing methods. Finally, comprehensive life cycle and health risk studies are needed: while Ti_3_C_2_T_*x*_ appears non-toxic at low doses, manufacturing emissions and material disposal must be managed responsibly. In summary, MXene-based materials demonstrate clear advantages in oil separation, combining ultra-high flux, selectivity, and multifunctionality, and hold great promise for scalable oil-spill and wastewater remediation. Realizing this potential will require concerted efforts to improve stability, develop eco-friendly synthesis and recycling methods, and integrate MXene systems into modular treatment processes. With ongoing innovations in materials design and green engineering, MXenes may soon transition from laboratory prototypes to field-ready remediation technologies.

## 7. Future recommendations

Green and scalable synthesis methods: Develop eco-friendly fabrication routes for MXenes, such as fluorine-free etching (alkali or molten-salt methods), electrochemical exfoliation, and recyclable etchant systems. Coupling MXene production with renewable energy (solar- or waste-heat-powered reactors) can dramatically reduce the carbon footprint. Scalable techniques (roll-to-roll deposition, continuous film casting, or 3D printing of MXene composites) should be prioritized to enable industrial-scale membrane and sorbent manufacturing.

Multifunctional photothermal and energy-recovering designs: Explore MXene-based platforms that harvest and utilize energy during separation. For instance, integrate broadband light-absorbing MXenes into membranes or sponges to heat and thin viscous oils under sunlight, enhancing flux and enabling passive self-cleaning. Combine MXene membranes with photocatalysts or electrodes to simultaneously purify water and generate usable energy (hydrovoltaic power or steam), leading to all-in-one decontamination devices that operate with minimal external energy input.

AI and computational materials design: leverage modeling and machine learning to guide MXene material optimization. Use first principles and molecular dynamics simulations to predict how different MXene chemistries, layer spacings, and surface terminations affect oil/water transport and fouling. Apply high-throughput computational screening and AI algorithms to discover novel MXene compositions (beyond Ti_3_C_2_) with tailored hydrophilicity or selectivity. Integrate experimental data and simulations *via* active learning to iteratively refine MXene filter designs for optimal performance and durability.

Novel MXene compositions and surface functionalities: investigate emerging MXene families and engineered terminations for enhanced separation. Synthesize and test nontraditional carbides/nitrides (*e.g.* Nb_2_C, Mo_2_C, V_2_C, double-layer MXenes) to exploit different chemical affinities, conductivities, or catalytic properties. Introduce atypical surface groups (–Cl, –S, –Se) through alternative etchants or post-treatment to adjust wettability and oil repellency. Such chemical innovation could yield MXene filters that switch wettability under stimuli or intrinsically resist chemical fouling.

Antifouling and self-cleaning architectures: design MXene surfaces and coatings that actively resist oil adhesion. Incorporate superhydrophilic/underwater-oleophobic nanostructures (*e.g.* zwitterionic polymers, TiO_2_, or ultrafine porosity) into MXene layers to form a slippery water barrier. Develop photocatalytic or photothermal self-cleaning strategies, for example, MXene membranes that heat or generate reactive species under light to degrade fouling oils. Optimize mechanical regeneration (backwashable channels and elastic composites) to enable multiple reuse cycles without performance loss.

Magnetic and stimuli-responsive sorbent systems: advance MXene-based sorbents that respond to external fields or signals. Continue integrating magnetic nanoparticles (Fe_3_O_4_) or shape-memory components into MXene sponges and aerogels to allow remote actuation and easy recovery. Explore electro-responsive or pH-responsive MXene composites that change wettability on demand, enabling the dynamic separation of oil-in-water and water-in-oil emulsions in a single unit. These smart materials could enable adaptive field-scale cleanup tools and real-time process control.

Comprehensive environmental and safety assessments: establish rigorous life cycle and toxicological evaluations of MXene-based systems. Conduct ecosystem-level studies of MXene degradation products and chronic exposure risks. Develop standards for MXene reuse and nanomaterial containment (*e.g.*, recovery of spent adsorbents and solvent reclamation). Ensure that applications prioritize MXene use cases where benefits outweigh production impacts and implement closed-loop recycling of MXene filters to minimize waste.

Integration with digital monitoring and control: couple MXene separation devices with smart sensors and IoT technologies. Embed flow, pressure, and fouling sensors into MXene membrane modules for real-time performance tracking. Utilize machine learning to predict maintenance needs and optimize operating conditions (*e.g.*, trigger light cleaning or backwashing when fouling is detected). Digital integration will be critical for deploying MXene systems in industrial facilities or *in situ* oil spill responses, enabling automated, adaptive remediation.

## Conflicts of interest

The author declares no conflicts of interest.

## Data Availability

No primary research results, software or code have been included and no new data were generated or analyzed in this review.
